# The Transcriptional Activator Krüppel-like Factor-6 Is Required for CNS Myelination

**DOI:** 10.1371/journal.pbio.1002467

**Published:** 2016-05-23

**Authors:** Benjamin M. Laitman, Linnéa Asp, John N. Mariani, Jingya Zhang, Jia Liu, Setsu Sawai, Candice Chapouly, Sam Horng, Elisabeth G. Kramer, Nesanet Mitiku, Hannah Loo, Natalie Burlant, Xiomara Pedre, Yuko Hara, German Nudelman, Elena Zaslavsky, Young-Min Lee, David A. Braun, Q. Richard Lu, Goutham Narla, Cedric S. Raine, Scott L. Friedman, Patrizia Casaccia, Gareth R. John

**Affiliations:** 1 Friedman Brain Institute, Icahn School of Medicine at Mount Sinai, New York, New York, United States of America; 2 Corinne Goldsmith Dickinson Center for Multiple Sclerosis, Icahn School of Medicine at Mount Sinai, New York, New York, United States of America; 3 Department of Neurology, Icahn School of Medicine at Mount Sinai, New York, New York, United States of America; 4 Department of Neuroscience, Icahn School of Medicine at Mount Sinai, New York, New York, United States of America; 5 Department of Medicine, Icahn School of Medicine at Mount Sinai, New York, New York, United States of America; 6 Systems Biology Center, Icahn School of Medicine at Mount Sinai, New York, New York, United States of America; 7 Pediatrics, Cincinnati Childrens’ Hospital, Cincinnati, Ohio, United States of America; 8 School of Medicine, Case Western Reserve University, Cleveland, Ohio, United States of America; 9 Department of Pathology, Albert Einstein College of Medicine, Bronx, New York, United States of America; UNITED STATES

## Abstract

Growth factors of the gp130 family promote oligodendrocyte differentiation, and viability, and myelination, but their mechanisms of action are incompletely understood. Here, we show that these effects are coordinated, in part, by the transcriptional activator Krüppel-like factor-6 (Klf6). Klf6 is rapidly induced in oligodendrocyte progenitors (OLP) by gp130 factors, and promotes differentiation. Conversely, in mice with lineage-selective *Klf6* inactivation, OLP undergo maturation arrest followed by apoptosis, and CNS myelination fails. Overlapping transcriptional and chromatin occupancy analyses place Klf6 at the nexus of a novel gp130-Klf-importin axis, which promotes differentiation and viability in part via control of nuclear trafficking. Klf6 acts as a gp130-sensitive transactivator of the nuclear import factor importin-α5 (Impα5), and interfering with this mechanism interrupts step-wise differentiation. Underscoring the significance of this axis in vivo, mice with conditional inactivation of gp130 signaling display defective Klf6 and Impα5 expression, OLP maturation arrest and apoptosis, and failure of CNS myelination.

## Abstract 2

During the development of white matter in the central nervous system, the transcription factor Klf6 coordinates pro-myelinating effects of gp130-Stat3 signaling, and promotes oligodendrocyte differentiation in part via control of nuclear trafficking.

## Author Summary

Myelin, a membranous sheet that surrounds the axons of neurons and facilitates the transmission of nerve impulses, is formed by oligodendrocytes in the central nervous system (CNS), and Schwann cells in the peripheral nervous system (PNS). Failure to myelinate, or demyelination, produces the symptoms of serious diseases such as leukodystrophies and multiple sclerosis. This study identifies a novel and essential regulator of myelin formation in the CNS: the transcriptional activator Krüppel-like factor-6 (Klf6). We show that Klf6 acts as a control point through which pro-myelinating signals regulate the development of oligodendrocytes from oligodendrocyte progenitor cells. Growth factors of the gp130 family are known to promote differentiation and survival of oligodendrocytes, via activation of the transcription factor Stat3, but the mechanistic details of how they do it remain unclear. Our findings show that Klf6 is induced in oligodendrocyte progenitors by gp130 growth factors, and promotes differentiation. Furthermore, oligodendrocyte progenitor cells in which Klf6 is inactivated do not mature, fail to form myelin, and eventually die. We also uncover that upon gp130 signaling, Klf6 activates the nuclear import factor importin-α5 (Impα5), a key controller of trafficking into the nucleus in the CNS. Notably, inactivation of Impα5 recapitulates the features of silencing Klf6, whereas Impα5 expression can partially rescue differentiation in Klf6-deficient oligodendrocyte progenitors. Finally, inactivation of Stat3 in oligodendrocyte progenitors produces defective Klf6 and Impα5 expression and myelination failure, strongly resembling the features produced by Klf6 inactivation. Collectively, these results establish a connection between gp130-Stat3 signaling and importin-based control of nuclear trafficking, which is essential for myelination in the CNS, and places Klf6 as a key coordinator of these events.

## Introduction

Myelin is essential for efficient nerve impulse transmission [1], and myelination failure, or demyelination, produces the symptoms of leukodystrophies and multiple sclerosis (MS) [2,3]. Remyelination restores conduction in MS, but is inefficient and fails in the latter stages of the disease [4]. Therapies that promote myelin formation are not yet available. A key goal in leukodystrophy and MS research is their successful translation to the clinic.

Within the central nervous system (CNS), myelin is formed by oligodendrocytes (OL), which differentiate from specified progenitors (OLP) [5]. The number of OL available for myelination is dependent on their viability and proliferation. Intrinsic transcriptional programs drive oligodendrocyte development and are influenced by extrinsic pro-myelinating or inhibitory cues [5]. Extrinsic pathways promoting differentiation include those triggered by PI3kinase-mTOR, Erk1/2, gp130-Stat3, and thyroid hormone (T3)/RXR signaling, and some of these also support viability [6–11]. Conversely, inhibitory cues for differentiation include those driven by Bmp and canonical Notch and Wnt signaling [5,12]. However, the mechanisms by which extrinsic signals influence myelination are incompletely characterized. Elucidating these mechanisms may identify key control points in oligodendrocyte development, manipulation of which may be used to promote myelin formation and repair.

Here, we now show that the pro-myelinating effects of gp130-Stat3 signaling are coordinated in part by Krüppel-like factor-6 (Klf6), a transcriptional activator of the Klf/Sp family. Members of this family are characterized by three C_2_H_2_-type zinc fingers that recognize GC-rich consensus motifs, and they act in combination to produce additive, synergistic, or differential outcomes [13]. Klf1, Klf4, and Klf5 have been shown to regulate hematopoietic, epidermal, and cardiovascular differentiation [14–16], and in different settings Klf4 also promotes stem cell maintenance [17]. Klf6 has been characterized as a tumor suppressor in prostate, ovary, liver, and gut [18], and Klf4, Klf5, and Klf6 are also implicated as regulators of viability [13]. In the CNS, Klf4 and Klf7 have been shown to regulate neuronal morphogenesis [19,20]. Klf15 has been found to promote astrocyte differentiation [21], and in oligodendrocytes, Klf9 has been identified as a T3-sensitive regulator of myelin repair. *Klf9*^*-/-*^ mice show delayed CNS remyelination, although they display no clear developmental myelination defects [22].

Our data identify Klf6 as required for developmental CNS myelination, and show that it coordinates pro-myelinating effects of the gp130-Stat3 pathway. Moreover, our findings suggest that gp130-Stat3-Klf6 signaling promotes viability and differentiation in part via control of nuclear trafficking. Klf6 acts as a gp130-sensitive transactivator of the nuclear import factor importin-α5 (Impα5), and interfering with this mechanism interrupts step-wise differentiation, with subsequent apoptosis. Underscoring the significance of the gp130-Klf-importin axis in myelination in vivo, our results show that mice with conditional inactivation of *Klf6* or canonical gp130-Stat3 signaling in the oligodendrocyte lineage display defective Impα5 regulation, maturation arrest followed by apoptosis, and failure of CNS myelination.

## Results

### Klf6 Is Induced by Extrinsic Pro-myelinating gp130-Stat3 Signaling in Oligodendrocyte Progenitors

**Fig 1:**
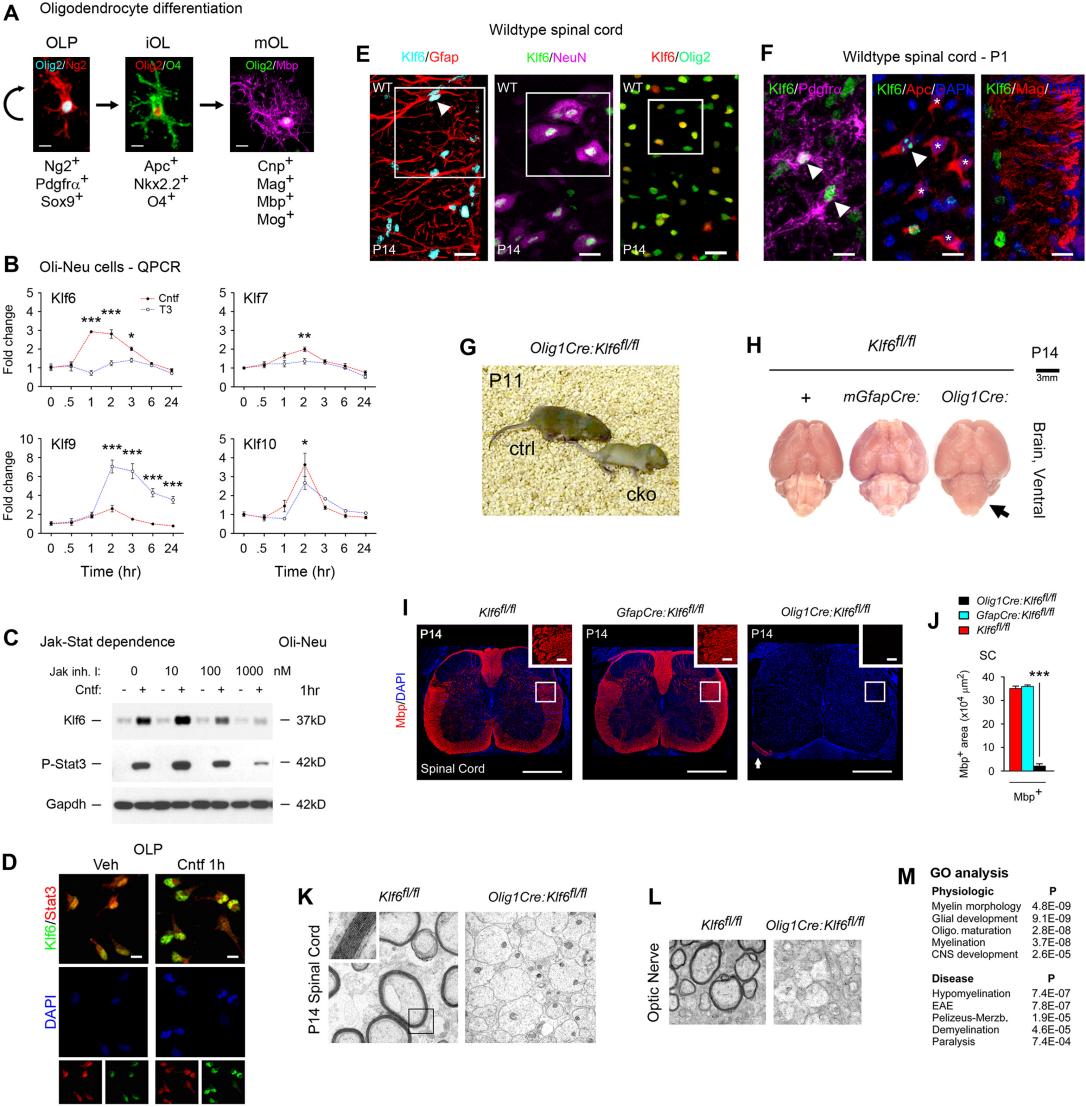
Klf6 is induced by gp130-Stat3 signals and is required for CNS myelination. **(A)** Stage-specific markers in differentiation. Following specification to the Olig2^+^ oligodendrocyte lineage, oligodendrocyte progenitors (OLP) express Ng2, Sox9, and Pdgfrα. Pro-myelinating signals induce differentiation to immature oligodendrocytes (iOL), marked by co-expression of Nkx2.2 and acquisition of Apc and O4. These undergo terminal differentiation to mature OL, which express myelin proteins. **(B)** Klf/Sp family responses to the pro-myelinating factors Cntf (100ng/ml) and T3 (40ng/ml), as determined by quantitative PCR (qPCR). **(C)** Immunoblotting for Klf6 in Oli-neu cultures pretreated with 0–1,000 nM Jak Inhibitor I 2 h, then exposed to 100 ng/ml Cntf 1 h. **(D)** Confocal imaging for Klf6 and the gp130 effector Stat3 in mouse OLP exposed to 100 ng/ml Cntf or vehicle 1 h. Cntf upregulation of Klf6 is associated with translocation to the nucleus, where it colocalizes with Stat3. **(E,F)** Klf6 expression visualized via confocal imaging in vivo. **(E)** In the postnatal CNS (spinal cord shown), immunoreactivity is heterogeneous in Olig2^+^ cells and more homogeneous in astrocytes (Gfap^+^) and neurons (NeuN^+^). Boxes and arrows highlight representative cells. **(F)** Klf6 is highly expressed (arrowheads) in OLP (left panel). In contrast, expression is lower (arrowhead) or undetectable (asterisks) in more mature Apc^+^ iOL (center panel), and Klf6 is not seen in mature Mag^+^ cells (right panel). **(G)** Still from S1 Video comparing a P11 *Olig1Cre*:*Klf6*^*fl/fl*^ mutant (cko, right) with sex-matched *Klf6*^*fl/fl*^ littermate (ctrl, left). The mutant is ataxic. **(H)** CNS white matter tracts in P14 *Olig1Cre*:*Klf6*^*fl/fl*^ mice display hypomyelination (arrowed), whereas *mGfapCre*:*Klf6*^*fl/fl*^ mice and *Klf6*^*fl/fl*^ littermate controls show no gross abnormalities. Brains are shown at the same magnification (scale bar, upper right). See S1 Fig. **(I,J)** Confocal analysis of lumbar spinal cords of P14 *Olig1Cre*:*Klf6*^*fl/fl*^ and *mGfapCre*:*Klf6*^*fl/fl*^ mice and *Klf6*^*fl/fl*^ controls. Myelin proteins are almost absent from *Olig1Cre*:*Klf6*^*fl/fl*^ spinal cord, whereas the peripheral nervous system (PNS) appears normal (arrowed). See S2 and S3 Figs. **(K,L)** Electron micrographs of P14 *Olig1Cre*:*Klf6*^*fl/fl*^ and *Klf6*^*fl/fl*^ spinal cords and optic nerves. Almost no myelin sheaths are present in *Olig1Cre*:*Klf6*^*fl/fl*^ CNS samples. **(M)** Gene ontology of BeadArray profiling of P1 CNS from *Olig1Cre*:*Klf6*^*fl/fl*^ pups and sex-matched *Klf6*^*fl/fl*^ littermates. The five most significant results are shown for physiologic and disease relevance. See also S4 Fig. Data are mean ± SEM. Statistics: **(B,J)** ANOVA plus Bonferroni test, *p < 0.05, ** *p* < 0.01, *** *p* < 0.001. Data are representative of two to four mice per genotype (for confocal imaging data) or three mice per genotype (for electron microscopy data). Scale, **(A)** 5 μm, **(E)** 20 μm, **(F)** 10 μm, **(H)** 3 mm **(I)** 150 μm, inset 15 μm. Magnifications, **(K)** x5,000, inset x20,000, **(L)** x3,000. Individual values are in S1 Data.

In the developing CNS, oligodendrocyte lineage cells are Olig2^+^, and OLP co-express the proteoglycan Ng2/Cspg4, SRY-box transcription factor 9 (Sox9), and mitogen receptor Pdgfrα (Fig 1A). Differentiation to the postmitotic immature oligodendrocyte (iOL) stage is marked by loss of OLP markers, co-expression of the transcription factor Nkx2.2 [23], and induction of adenomatous polyposis coli (Apc) and O4 [5]. Subsequently, iOL undergo terminal differentiation to become mature oligodendrocytes (mOL), which are extensively arborized and express the myelin proteins Cnp, Mag, Mbp, and Mog [5].

Initial data suggesting a role for Klf6 in oligodendrocyte development came from screening for responses to pro-myelinating signals in the mouse OLP line Oli-neu [24] and primary OLP [25]. Amongst Klf family members known to be CNS-expressed, Klf6 and Klf7 were upregulated by the gp130 ligands Cntf and Lif, while Klf9 was sensitive to T3 [22] and Klf10 was upregulated by ligands for both pathways (Figs 1B and S1A). Klf6 displayed the most rapid and sustained response to gp130 factors, detectable by 1 h and persisting through 3 h. Cntf induction of Klf6 was dependent on Jak-Stat signaling, and resulted in nuclear translocation (Fig 1C and 1D). Based on these data, our attention was directed to Klf6 as a potential effector of pro-myelinating gp130 signals.

### Klf6 Is Downregulated during OLP Differentiation and Is Essential to Successful CNS Myelination

We examined Klf6 expression in the developing spinal cord, a well-characterized system for studying myelination [5]. Confocal imaging of early postnatal (P1–14) samples revealed heterogeneous expression by Olig2^+^ cells, and more homogeneous astrocytic and neuronal immunoreactivity (Fig 1E). The source of heterogeneity in the oligodendrocyte lineage was revealed by staining for stage-specific markers, which showed that Klf6 is downregulated during differentiation. Klf6 was highly expressed in OLP, whereas immunoreactivity was low or absent in more mature Apc^+^ iOL and was not observed in mature Mag^+^ cells (Fig 1F).

**Fig 2:**
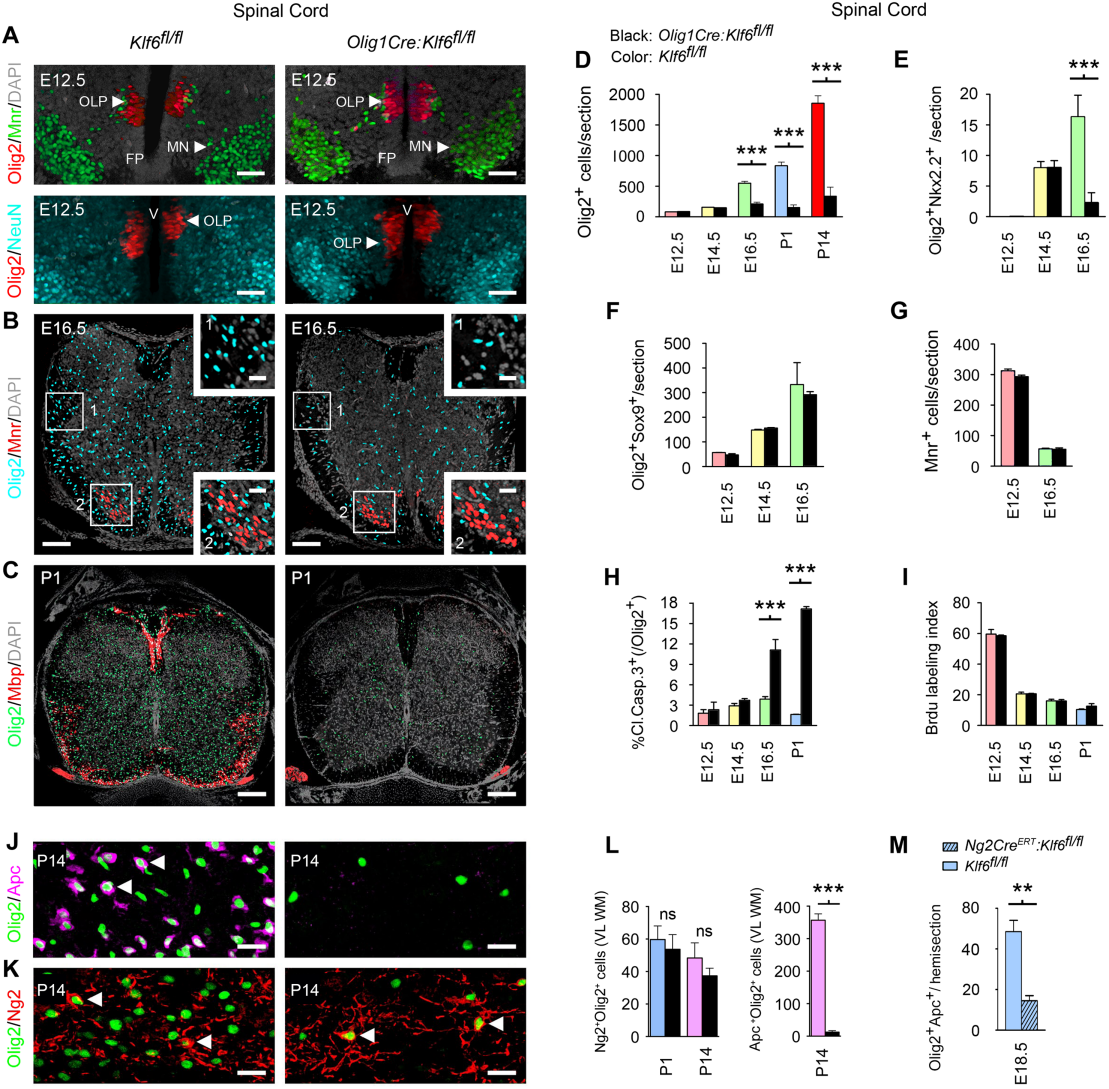
Conditional *Klf6* inactivation in vivo causes selective loss of differentiating oligodendrocytes. **(A–F)** Confocal and morphometric analysis of oligodendrocyte numbers in developing *Olig1Cre*:*Klf6*^*fl/fl*^ and control *Klf6*^*fl/fl*^ spinal cords. Panel **(A)** shows samples from E12.5, immunostained for Olig2 and either Mnr (upper panels) or NeuN (lower panels). At E12.5, Olig2^+^ numbers in *Olig1Cre*:*Klf6*^*fl/fl*^ and control samples are identical, and no differences are seen in neuronal markers. In controls, Olig2^+^ cell numbers then increase from E16.5 through P14, but no increase is seen in *Olig1Cre*:*Klf6*^*fl/fl*^ samples **(B–D)**. Areas outlined in panel **(B)** are shown at higher magnification, inset. See also S4B Fig. At P1, myelin proteins are expressed in controls, but not in *Olig1Cre*:*Klf6*^*fl/fl*^ spinal cords **(C)**. **(E,F)** Analysis of stage-specific markers. Early events in differentiation, such as Nkx2.2 co-expression at E14.5, occur normally in *Olig1Cre*:*Klf6*^*fl/fl*^ mice, but differentiating cells are selectively lost at subsequent timepoints **(E)**. In contrast, numbers of Olig2^+^Sox9^+^ OLP remain identical to controls **(F)**. There are no changes in Mnr^+^ motor neurons, which share the same origin as ventral OLP in the pMN domain (**A** upper panels, **B, G**). **(H,I)** Selective loss of differentiating cells is associated with increased apoptosis **(H)**, but OLP proliferation is unaffected **(I)**. **(J–L)** Postnatal stage-specific analysis confirms absence of differentiating (Apc^+^) oligodendrocytes from *Olig1Cre*:*Klf6*^*fl/fl*^ mice **(J,L)**, whereas OLP (Olig2^+^Ng2^+^) numbers are comparable to controls **(K,L)**. Representative cells are arrowed. **(M)** Analysis of *NG2creER*^*–*^:*Klf6*^*fl/fl*^ mice, in which *Klf6* inactivation is inducibly targeted to OLP. See also S4C and S4D Fig. Similar to *Olig1Cre*:*Klf6*^*fl/fl*^ embryos, these mice also display selective loss of differentiating oligodendrocytes. Data are mean ± SEM. Statistics, **(D–I,L)** ANOVA plus Bonferroni post test, **(M)** Student’s *t* test, ***p < 0.001. Scale: **(A,B)** 100 μm, inset 20 μm, **(C)** 250 μm, **(J,K)** 10 μm. Data shown are from lumbar sections of two to six mice per genotype per timepoint. Thoracic sections showed compatible findings. Individual values are in S1 Data.

To determine the functional significance of Klf6 in oligodendrocyte development, we used conditional inactivation in vivo. Importantly, these studies revealed that Klf6 is required for CNS myelination. To inactivate *Klf6* throughout the lineage, we crossed a floxed allele [26] with *Olig1Cre* [27] to generate experimental *Olig1Cre*:*Klf6*^*fl/fl*^ mice and three control genotypes (*Olig1Cre*:*Klf6*^*fl/+*^, *Klf6*^*fl/fl*^, *Klf6*^*fl/+*^) (S1B and S1C Fig). Findings were confirmed using an alternate *Cre* cassette (see Fig 2). We further compared the results of astrocyte-selective inactivation, generated using *Klf6*^*fl*^ and *mGfapCre* (S1B and S1C Fig) [28].

Initial results came from *Olig1Cre*:*Klf6*^*fl/fl*^ and *mGfapCre*:*Klf6*^*fl/fl*^ lines. Specificity and efficiency of inactivation in each line were confirmed by confocal imaging for lineage markers (S1D Fig), and data from these studies were compatible with previously reported findings for both cassettes [27,28]. In each breeding program, experimental pups appeared normal at birth, but from the second postnatal week, *Olig1Cre*:*Klf6*^*fl/fl*^ pups displayed progressive tremor and ataxia (Fig 1G and S1 Video). Examination of gross pathology at P14 revealed severe CNS hypomyelination in *Olig1Cre*:*Klf6*^*fl/fl*^ mice (Fig 1H). No *Olig1Cre*:*Klf6*^*fl/fl*^ pups survived the third postnatal week, death occurring due to seizures consequent to the myelination defect. In contrast, *mGfapCre*:*Klf6*^*fl/fl*^ pups showed normal weight gain, behavior, and survival, and a normal-appearing CNS (Fig 1H), suggesting a non-critical role for Klf6 in astrocyte development.

We confirmed hypomyelination using confocal imaging. At P14, myelin proteins were present in controls but absent in *Olig1Cre*:*Klf6*^*fl/fl*^ samples in all tracts examined, including spinal cord, hindbrain, cerebellum, corpus callosum, and optic nerves (Figs 1I 1J, S2A, S2B and S3A–S3D). In contrast, we detected no neuronal or astrocytic pathology (S2C, S2D, S3E and S3F Figs). We validated these data using light and electron microscopy, which confirmed selective loss of CNS myelin sheaths (Figs 1K, 1L, S2E–S2K and S3G–S3I), whereas peripheral nerves were myelinated normally (Figs 1I and S2J). Myelination failure persisted for the lifespan of *Olig1Cre*:*Klf6*^*fl/fl*^ mice, and was not associated with axonal pathology, even shortly prior to death (S2K Fig).

### Oligodendrocyte Transcripts Are Selectively Lost from the CNS of *Olig1Cre*:*Klf6*^*fl/ffl*^ Mice

BeadArray transcriptional profiling confirmed selective loss of oligodendrocyte transcripts from the CNS of *Olig1Cre*:*Klf6*^*fl/fl*^ mice. Comparison of *Olig1Cre*:*Klf6*^*fl/fl*^ and *Klf6*^*fl/fl*^ CNS at P1 identified 51 differentially expressed transcripts (Q < 0.05), all but one of which were reduced in *Olig1Cre*:*Klf6*^*fl/fl*^ samples (S4A Fig). Using a published database [29], we confirmed all reduced transcripts as oligodendrocyte lineage-expressed, and 26 as lineage-specific. Gene ontology analysis using Ingenuity software mapped the cohort to myelin morphology, oligodendrocyte maturation, and myelination. Disease association identified hypomyelination, the MS model EAE, Pelizeus-Merzbacher leukodystrophy, and demyelination (Fig 1M).

### Selective Loss of Differentiating Oligodendrocytes from the CNS of *Olig1Cre*:*Klf6*^*fl/fl*^ Embryos

Transcriptional profiling of entire CNS data did not distinguish between changes in expression in living cells, versus global changes in expression due to loss of the cells that normally express the transcript. Therefore, to identify underlying cellular events, we used confocal imaging of developing thoracolumbar spinal cord (Figs 2 and S4). At E12.5, following OLP specification, through expansion at E14.5, Olig2^+^ cell numbers in *Olig1Cre*:*Klf6*^*fl/fl*^ samples were identical to controls (Fig 2A and 2D). Early events in differentiation, such as Nkx2.2 co-expression at E14.5, occurred normally (Fig 2E). However, while Olig2^+^ cells then increased in controls, no increase occurred in *Olig1Cre*:*Klf6*^*fl/fl*^ mice (Fig 2B–2D). Remaining Olig2^+^ cells displayed a typical spatial distribution, indicating normal migration and dorsal specification (Fig 2B and 2C). Myelin proteins were seen from P1 in controls, but not in *Olig1Cre*:*Klf6*^*fl/fl*^ samples (Fig 2C). Heterozygotes showed no pathology or behavioral defects (S4B Fig).

Critically, further analysis revealed that the reduction in Olig2^+^ cells in *Olig1Cre*:*Klf6*^*fl/fl*^ samples was caused by selective loss of differentiating Olig2^+^Nkx2.2^+^, or Olig2^+^Apc^+^, cells (Fig 2E). In contrast, numbers of Olig2^+^Sox9^+^ (or Olig2^+^Ng2^+^) OLP were identical to controls (Fig 2F). There were no changes in NeuN^+^ neurons, or in Mnr^+^ motor neurons, which share the same origin as ventral OLP in the pMN domain (Fig 2A, 2B and 2G) [27]. Loss of differentiating cells occurred via apoptosis (Fig 2H). In contrast, OLP proliferation was normal (Fig 2I). Postnatal analysis confirmed persistent absence of maturing oligodendrocytes in *Olig1Cre*:*Klf6*^*fl/fl*^ mice, whereas OLP numbers remained equal to controls (Fig 2J–2L).

We validated specificity of the hypomyelination phenotype using a second Cre driver, *NG2creER*^*TM*^, which produces inducible recombination in OLP in vivo [30]. *Cre* was induced at E12.5 and embryos harvested at E18.5. Olig2^+^Apc^+^ oligodendrocytes were selectively lost in *NG2creER*^*TM*^:*Klf6*^*fl/fl*^ samples (Figs 2M, S4C and S4D).

### Klf6 Promotes Oligodendrocyte Differentiation and Viability

**Fig 3:**
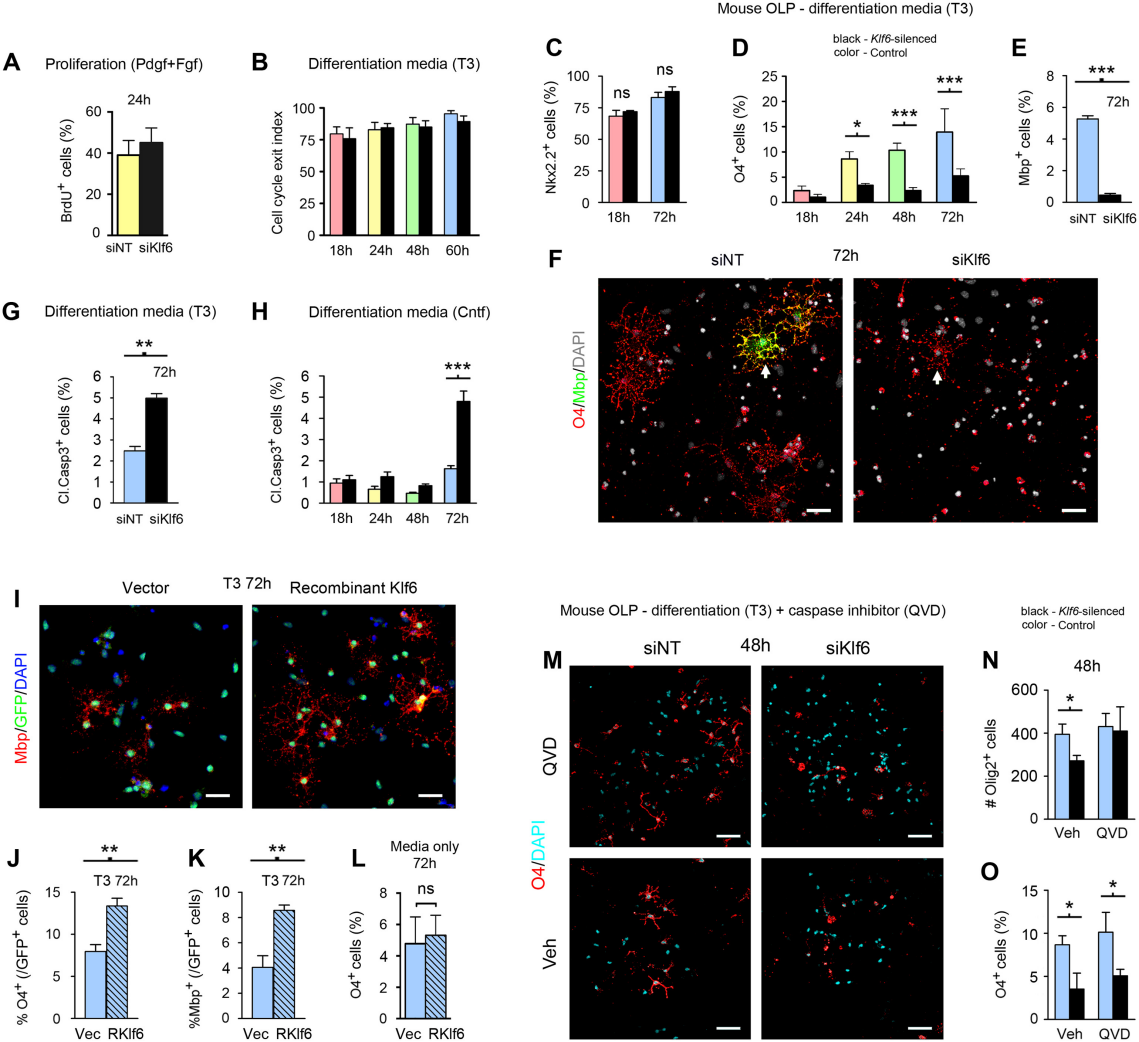
Klf6 is required for differentiation regardless of the initiating stimulus. **(A–H)** Results of confocal analysis of primary mouse OLP subjected to *Klf6* silencing as described in Materials and Methods, and treatments indicated. *Klf6* silencing has no effect on active proliferation, shown by BrdU immunoreactivity in proliferating cultures **(A)**, nor on cell cycle exit index (Ki67^-^BrdU^+^/BrdU^+^) in differentiating OLP cultures **(B)**. **(C–F)** Early events in differentiation, such as Nkx2.2 co-expression, occur normally in *Klf6*-silenced OLP differentiated with either 40 ng/ml T3 or 100 ng/ml Cntf **(C)**. However, expression of markers of subsequent stages of differentiation, such as O4 and Apc, is delayed in silenced cultures compared with non-targeting (NT) controls, and these cultures fail to mature to Mbp^+^ mOL **(D–F)**. See also S4G Fig. These changes are associated with increased apoptosis **(G,H)**. **(I–L)** Confocal imaging analysis of primary mouse OLP nucleofected with GFP-labeled *Klf6*-overexpression construct or GFP vector control, and either left untreated or exposed to 40 ng/ml T3 for 72 h. Klf6 overexpression accelerates differentiation triggered by a pro-myelinating signal (T3), as measured by induction of O4 and Mbp **(I–K)**. However, Klf6 overexpression alone does not initiate differentiation **(L)**. **(M-O)** Confocal imaging analysis of *Klf6*-silenced and NT control mouse OLP pretreated with 2 μM caspase inhibitor Q-VD-OPh or vehicle for 2 h, then differentiated with T3 for 48 h. Apoptosis is almost absent from cultures exposed to the inhibitor, and Olig2^+^ cell numbers in *Klf6*-silenced and control conditions are almost identical **(M,N)**. However, rescue of viability does not restore differentiation, as measured by the percentage of O4^+^ cells **(M,O)**. Statistics, **(A,E,G,J,K,L)** Student’s *t* test, **(B,C,D,H,N,O)** ANOVA plus Bonferroni post-test, *p < 0.05, **p < 0.01, ***p < 0.001. Data are representative of at least three independent studies in separate cultures. Individual values are in S1 Data.

To investigate cellular mechanism in more detail, we used mouse OLP cultures and the Oli-neu line (Figs 3 and S4). Initially, we examined Klf6 expression during differentiation. In Oli-neu cells and OLP, exposure to Cntf or T3 produced comparable rates of differentiation. In Cntf- but not T3-treated cultures, this was associated with Klf6 upregulation (S4E Fig). In both sets of cultures, Klf6 then decreased to baseline, coincident with differentiation (S4F Fig) and matching the pattern in developing white matter (see Fig 1F).

We tested the functional impact of *Klf6* silencing in mouse OLP in vitro. Cultures were nucleofected with *Klf6* siRNA or non-targeting (NT) control (see S4G Fig), then exposed to pro-myelinating signals and harvested for confocal imaging at 24 h intervals. Silencing produced no effect on the rate of proliferation in OLP cultures prior to differentiation (Fig 3A), nor on the rate of cell cycle exit index (Ki67^-^BrdU^+^/BrdU^+^) in differentiating cultures (Fig 3B). Moreover, early events in differentiation, such as Nkx2.2 co-expression, occurred normally in *Klf6*-silenced cultures, and the percentage of these cells in silenced cultures and controls remained similar, compatible with our findings from *Olig1Cre*:*Klf6*^*fl/fl*^ mice (Fig 3C and see Fig 2E). However, expression of markers of subsequent stages of differentiation, such as O4 and Apc, was delayed in silenced cultures, and this effect was statistically significant from 24 h (Fig 3D and 3F). Moreover, silenced cultures displayed failure of terminal differentiation to Mbp^+^ mOL (Fig 3E and 3F). These effects were associated with increased apoptosis, which was prominent by 72 h, and, interestingly, these outcomes occurred whether differentiation was induced using Cntf or T3 (Fig 3G and 3H).

These data suggested that while Klf6 is regulated by some pro-myelinating factors (Cntf, Lif) but not others (T3), its presence is required for differentiation, regardless of the initiating stimulus. *Klf6* inactivation has no effect on OLP proliferation or cell cycle exit, or on the earliest stages of differentiation. The impact of its loss is first observed during the latter phases of differentiation to the O4^+^Apc^+^ iOL stage, and subsequent progression to terminal maturation fails. These events are associated with apoptosis of affected cells. Thus, Klf6 appears to function in the early stages of differentiation, after cell cycle exit but before more terminal stages of maturation.

### Klf6 Overexpression Accelerates Differentiation

We tested the impact of Klf6 gain of function on differentiation in vivo. We nucleofected OLP with a GFP-tagged Klf6 overexpression vector or GFP-empty control, then exposed to T3 or vehicle control and harvested at 24 h intervals for confocal imaging analysis. Importantly, increasing Klf6 in differentiating cultures accelerated differentiation, as measured by induction of O4 and Mbp (Fig 3I–3K). However, Klf6 overexpression alone did not initiate differentiation, in that it did not trigger acquisition of differentiation markers in the absence of an extrinsic pro-myelinating signal (Fig 3L).

### Rescue of Viability in Klf6-Deficient Cultures Does Not Restore Differentiation

Since we detected apoptosis of differentiating cells in *Klf6*-silenced cultures, we tested whether Klf6 acts primarily on differentiation or viability, or independently on both. We grew *Klf6*-silenced and NT control mouse OLP in the presence of the caspase inhibitor Q-VD-OPh 2 μM or vehicle, then induced differentiation and harvested at 48 h. Apoptosis was almost absent in cultures exposed to the caspase inhibitor, and numbers of Olig2^+^ cells were almost identical (Fig 3M and 3N). However, inhibition of apoptosis in *Klf6-*silenced cultures did not rescue differentiation (Fig 3M and 3O).

These data suggested that Klf6 plays distinct roles in differentiation and viability. Our findings indicate that some pro-myelinating cues, such as gp130 signaling, use Klf6 to set the rate of differentiation. Others, such as T3, must use alternate means to achieve a similar end. Moreover, a basal level of Klf6 is required for differentiation regardless of the initiating trigger. Interestingly, these effects are compatible with roles proposed for gp130-Stat3 signaling on viability and maturation, in the oligodendrocyte lineage and more widely [10,11,31,32].

### Transcriptional Profiling Links Klf6 to Differentiation and Upstream gp130-Stat3 Signaling

**Fig 4:**
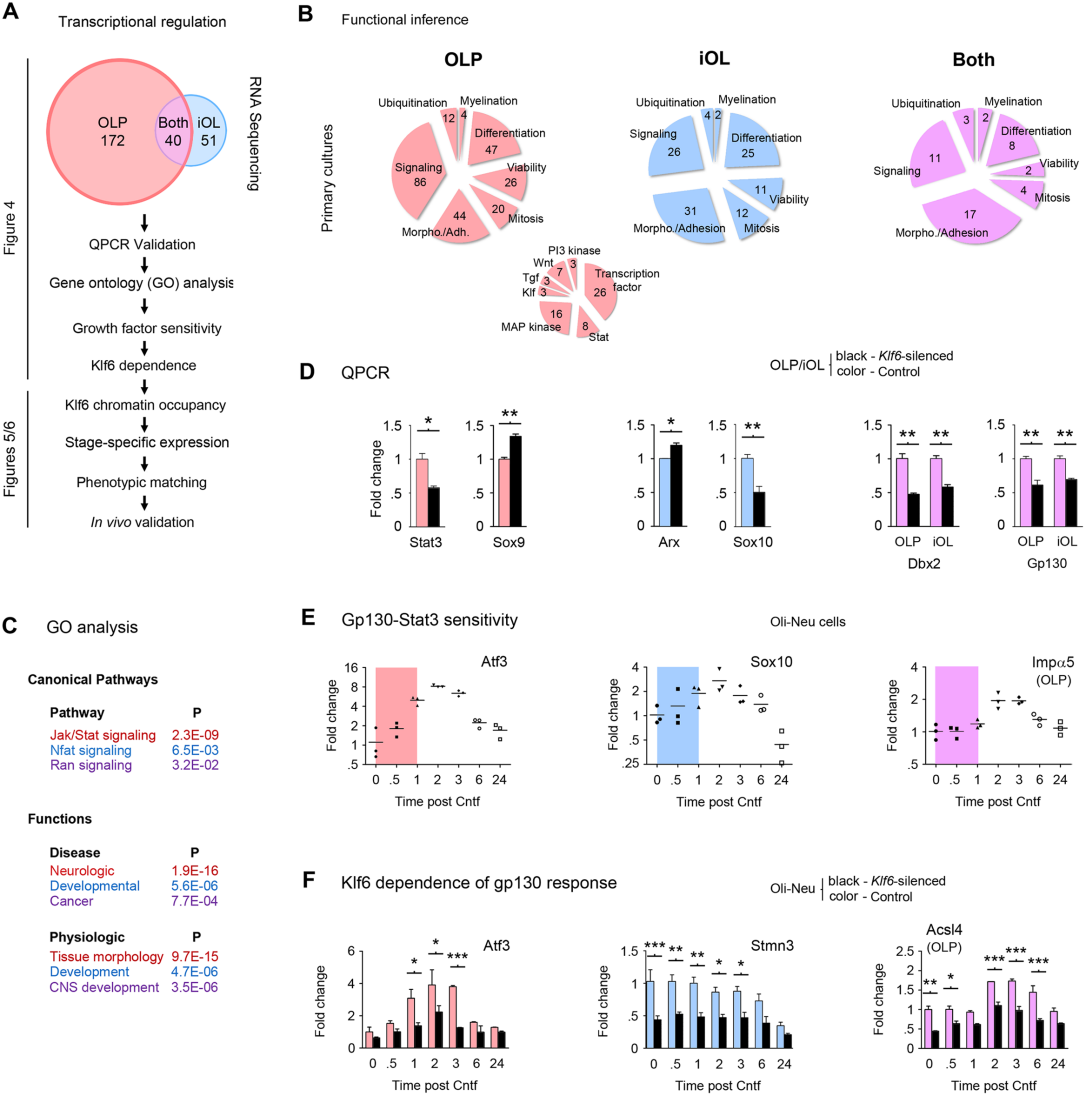
RNA sequencing identifies Klf6-dependence of gp130-driven transcriptional patterns. **(A)** Overview of approach used to define key Klf6 effectors. Initial RNA-seq analysis of primary mouse cultures identifies 212 unique Klf6-regulated transcripts in OLP and 91 in iOL, of which 40 are shared. See S1–S3 Tables and S5A and S5B Fig. **(B,C)** Results of functional inference **(B)** and GO analysis **(C)** of RNA-seq data, from primary OLP (red), iOL (blue), or both (purple). In **(B)**, numbers of Klf6-regulated genes are indicated for each function. Implicated signaling pathways in OLP are presented as a smaller Venn diagram, inset. **(D)** Examples of qPCR validation of RNA-seq data for select OLP, iOL, and shared genes. A larger cohort of validation data is presented in S5C Fig. **(E)** Gp130 sensitivity of select validated differentially expressed transcripts. Results are shown from Oli-neu cells treated with Cntf (100 ng/ml) for up to 24 h. Colored areas indicate the time period before peak response of Klf6 to Cntf. See also S5D Fig. **(F)** Klf6-dependence of Cntf-induced responses. qPCR analysis of *Klf6-*silenced and control Oli-neu cells treated with Cntf for up to 24 h. Cntf sensitivity of differentially expressed targets is blunted in *Klf6*-silenced samples. Note also that some genes are Cntf-independent but Klf6-dependent during differentiation. Data are mean ± SEM. Statistics, **(D)** Student’s *t* test, **(F)** Two-way ANOVA plus Bonferroni post-test, *p < 0.05, **p < 0.01, ***p < 0.001. Data are representative of two to three independent studies. RNA-seq data are presented in full in S1–S3 Tables and are available on the GEO website (http://www.ncbi.nlm.nih.gov/geo/) (Accession number GSE79245). Individual values for all other quantifications are in S1 Data.

To find important molecular mechanisms by which Klf6 exerts its effects, we initially used RNA sequencing (RNA-seq) to identify a full cohort of Klf6-regulated transcripts (Figs 4 and S5, S1–S3 Tables). This work compared transcript numbers in *Klf6-*silenced versus NT control primary mouse OLP, and O4^+^Apc^+^Mbp^-^ iOL differentiated with T3 for 18 h (S5A and S5B Fig). These studies identified 212 Klf6-regulated transcripts in OLP and 91 in iOL, of which 40 were shared (Fig 4A).

Analysis of this dataset supported a role for Klf6 in differentiation—almost half of the genes identified as Klf6-sensitive have been implicated previously as regulating differentiation and/or morphology (Fig 4B). In OLP, we identified the transcription factors Sox8, Sox9, and Sox21, which regulate oligodendrocyte and neural progenitor differentiation [33–35]. In iOL, Klf6 also regulated Sox10, which is required for OLP maturation [36]. Additionally, in OLP we identified the Wnt and Notch pathway components Wnt7b and Dact1, which limit differentiation (Fig 4B and S1 Table) [37,38], while in iOL, Klf6 regulated the homeobox transcription factors Arx and Dbx2, which are required for normal neural progenitor development (Fig 4B and S2 Table) [39,40].

RNA-seq data also suggested reciprocal interactions of Klf6 and the gp130-Stat3 pathway. We identified Klf6 regulation of the pathway components gp130, Stat-1 and -3, Jak1, and Socs2 and -3, suggesting that Klf6 exerts feedback on the pro-myelinating pathway that induces its expression (Fig 4B and S1 and S3 Tables). Gene ontology of the RNA-seq dataset also identified gp130 signaling as Klf6-regulated (Fig 4C). Collectively, these results suggested Klf6 as a coordinator that links gp130-Stat3 signaling and differentiation.

Intriguingly, RNA-seq data also suggested potential downstream effectors through which Klf6 might exert its actions. Notably, we identified Klf6 regulation in both OLP and iOL of Impα5, the major controller of transcription factor nuclear import in the adult CNS [41,42]. Importins are required for success of transcriptional programs, including those controlling differentiation and viability (Fig 4B and S3 Table) [41,43]. Thus, this finding suggested a potential effector mechanism by which Klf6 might regulate oligodendrocyte development.

We validated this dataset using qPCR screening of a gene subset in mouse OLP and Oli-neu cells. These studies confirmed that *Klf6* silencing resulted in downregulation of gp130 pathway components (Stat3, gp130), and transcripts associated with differentiation (Sox10, Dbx2) and nuclear import (Impα5), and upregulation of transcripts linked to progenitor maintenance (Arx, Sox9) (Figs 4D and S5C). Interestingly, although Sox9 helps to maintain Pdgfrα expression in progenitors [44], our data from silencing studies in vitro showed that *Klf6* inactivation (and associated increases in Sox9) was not associated with changes in OLP proliferation or cell cycle exit (see Fig 3A and 3B). Notably, qPCR validation also extended our RNA-seq findings in that it confirmed that Klf6 mediates responses to pro-myelinating gp130 signals. Half of validated genes identified as Klf6-regulated were also sensitive to gp130-Stat3 induction, including Sox10 and Impα5 (Figs 4E and S5D), and these responses were Klf6-dependent (Fig 4F). The remaining cohort was Klf6-dependent and Cntf-independent, suggesting regulation by basal Klf6 levels that existed in OLP prior to downregulation during differentiation. This latter cohort included several genes relevant to progenitor development and migration, including Arx, Dbx2, the semaphorin receptor plexin B3, and the cytoskeletal adaptor Stmn3, which has recently been linked to glioma motility (Figs 4F and S5D) [45].

### Analysis of Klf6 Chromatin Occupancy Identifies a gp130-Klf-Importin Signaling Axis

**Fig 5:**
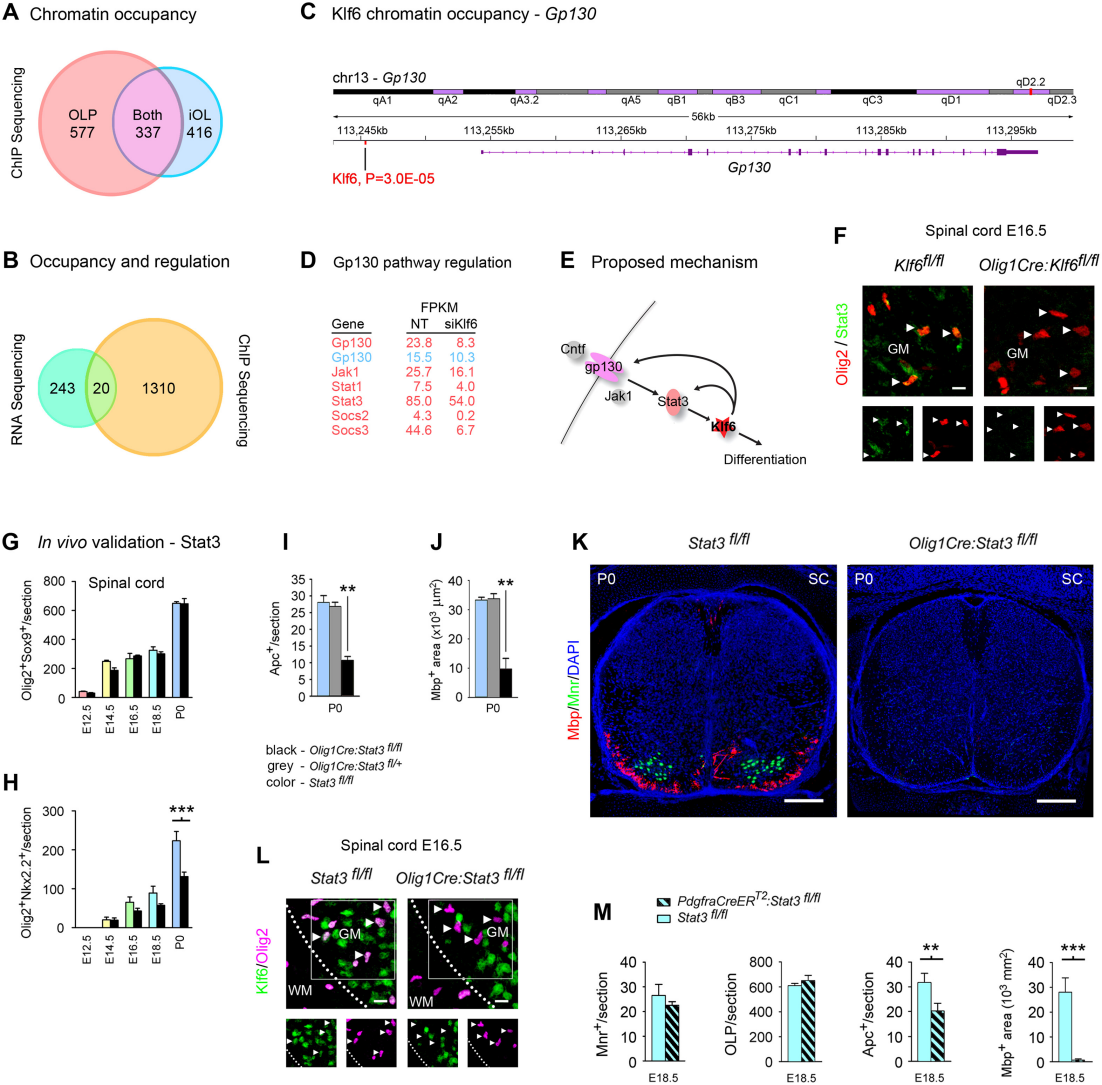
*Stat3* inactivation in vivo produces failure of myelination, similar to *Klf6* inactivation. **(A)** Graphical overview of genome-scale chromatin occupancy (ChIP-seq) data for Klf6 in primary mouse OLP and iOL. ChIP-seq analysis identifies 577 peaks of Klf6 chromatin binding within 20 kb of transcription start sites in OLP, 416 in iOL, and 337 that are shared. See also S4 Table. **(B)** Cross-referencing this ChIP-seq dataset to RNA-seq data in S1 and S2 Tables identifies 20 genes as prospective directly-regulated Klf6 effectors. This cohort is presented in S5 Table. **(C,D)** ChIP-seq identifies Klf6 binding to the promoter of the *gp130* gene **(C)**, which is also amongst multiple gp130-Stat3 signaling pathway components identified by RNA-seq as Klf6-regulated (**D**, and see S1–S3 Tables). These findings suggest that Klf6 exerts feedback upon the gp130-Stat3 pathway that drives its expression **(E)**. **(F)** Confocal images of spinal cords of E16.5 *Olig1Cre*:*Klf6*^*fl/fl*^ and *Klf6*^*fl/fl*^ control embryos labeled for Stat3 and Olig2. Areas illustrated are from grey matter (GM). Levels of Stat3 are reduced in the oligodendrocyte lineage in *Olig1Cre*:*Klf6*^*fl/fl*^ samples. Arrowheads mark representative cells, which are shown at higher magnification below. See also S6A Fig. **(G–M)** Confocal analysis of spinal cords from mice with *Stat3* inactivation targeted to OLP (*Olig1Cre*:*Stat3*^*fl/fl*^ and *PdgfracreER*^*TM*^:*Stat3*^*fl/fl*^). The oligodendrocyte phenotype of these mice resembles that produced by conditional *Klf6* inactivation (see Fig 2). At E12.5–14.5, OLP numbers in conditional *Stat3* mutants are comparable to *Stat3*^*fl/f*^ controls, and early events in differentiation occur normally **(G,H)**. However, selective loss of differentiating cells is seen from E16.5 onward **(H,I)**, and myelination is profoundly disrupted (**J,K**, see also S6B and S6C Fig). *Stat3* inactivation produced by *Olig1Cre* also results in loss of motor neurons, which share the same origin in the pMN domain (**K,** and see S6D Fig). **(L)** Spinal cords of E16.5 *Olig1Cre*:*Stat3*^*fl/fl*^ and control *Stat3*^*fl/fl*^ embryos, labeled for Klf6 and Olig2. The border between grey (GM) and white matter (WM) is marked, and representative cells are arrowed. Conditional *Stat3* inactivation produces defective Klf6 expression. **(M)** Conditional *Stat3* inactivation in OLP using *PdgfracreER*^*TM*^ results in selective failure of OLP differentiation and myelination. Motor neurons are unaffected. Data are mean ± SEM. Statistics, **(G–J)** ANOVA plus Bonferroni test, **(M)** Student’s *t* test, **p < 0.01, ***p < 0.001. Scalebars, **(F,L)** 5 μm, **(K)** 250 μm. Data are representative of two to six mice per genotype per time point. Results in **(F–M)** are from lumbar sections. ChIP-seq data are presented in full in S4 Table, RNA-seq and ChIP-seq intersect data are presented in full in S5 Table, and both datasets are available on the GEO website (http://www.ncbi.nlm.nih.gov/geo/) (Accession number GSE79245). Individual values for all other quantifications are in S1 Data.

To identify genes that are directly Klf6-controlled, we used chromatin immunoprecipitation and sequencing (ChIP-seq) to analyze Klf6 promoter occupancy, then cross-referenced the results to the RNA-seq dataset. ChIP-seq identified 914 sites of Klf6 binding to prospective control regions of annotated genes in OLP, and 753 in iOL, of which 337 were shared (Fig 5A and S4 Table). Superimposing the ChIP-seq and RNA-seq data identified an overlapping cohort of 20 genes. Notably, these included gp130 and Impα5 (Fig 5B and S5 Table).

**Fig 6:**
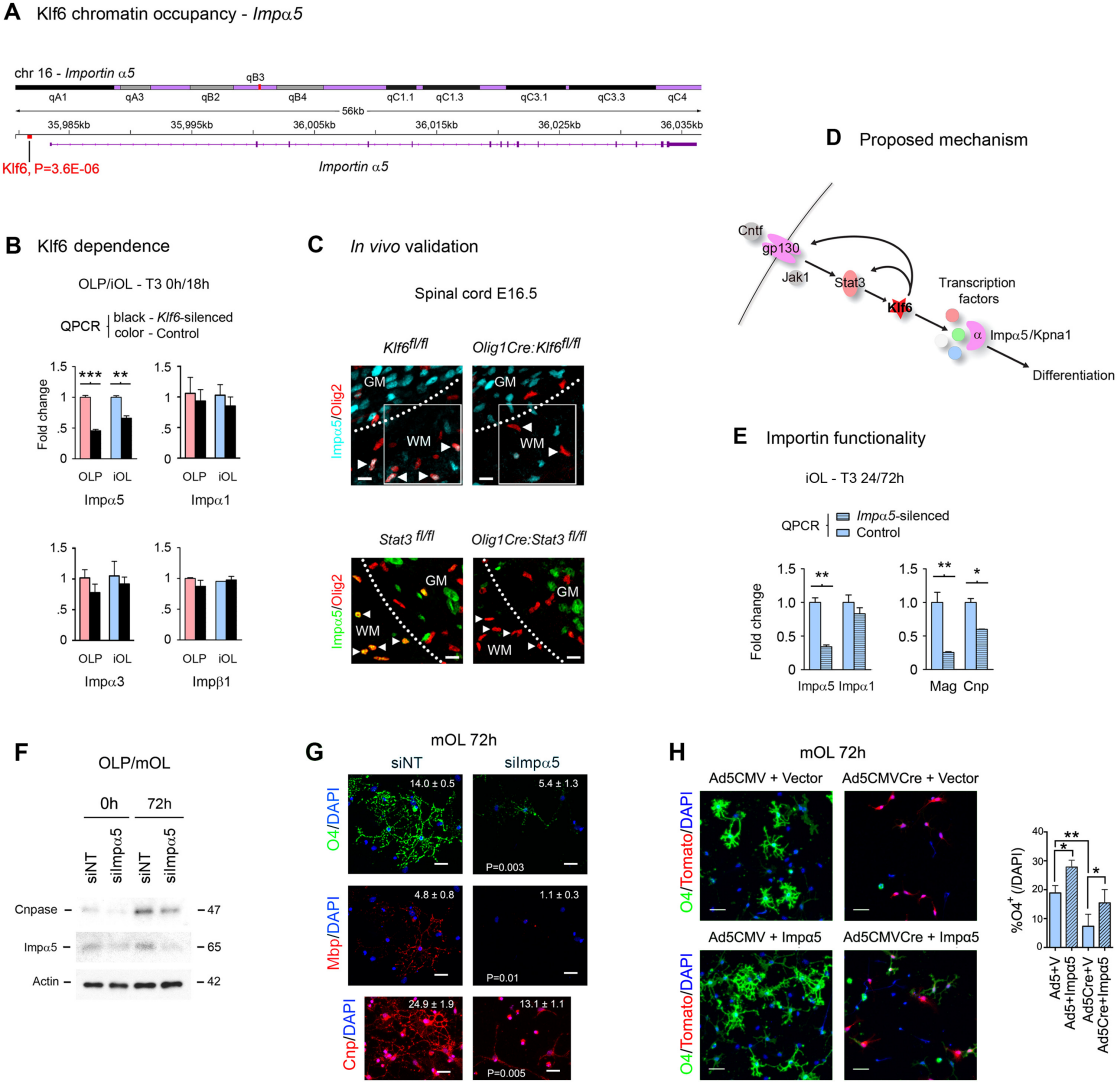
Klf6 promotes differentiation via transactivation of importin-α5. **(A–C)** ChIP-seq **(A)**, qPCR **(B)**, and confocal imaging data **(C)** identifying Klf6 regulation of the downstream importin effector Impα5. Data are also compatible with RNA-seq results in S1–S3 Tables. **(A)** ChIP-seq in primary mouse OLP and iOL demonstrates direct Klf6 binding to the promoter region of *Impα5*, which RNA-seq also identifies as Klf6-regulated (see S3 Table). **(B)** QPCR for importin family members in *Klf6-*silenced versus NT control primary mouse OLP, and O4^+^Apc^+^Mbp^-^ iOL differentiated with T3 for 18 h. Note that Klf6 control of Impα5 expression is selective. **(C)** Confocal imaging of lumbar spinal cords of E16.5 *Olig1Cre*:*Klf6*^*fl/fl*^ and *Klf6*^*fl/fl*^ control embryos (upper panels), and *Olig1Cre*:*Stat3*^*fl/fl*^ and Stat3^*fl/fl*^ control embryos (lower panels), showing reduced Impα5 expression in oligodendrocyte lineage cells in mice with conditional *Klf6* or *Stat3* inactivation. Arrowheads in white matter areas (outlined) mark representative cells. Collectively, findings in **(A–C)** are compatible with the hypothesis that gp130-driven Klf6 uses selective control of Impα5 to regulate oligodendrocyte development **(D)**. **(E–G)** qPCR, confocal imaging, and immunoblotting data from primary mouse OLP silenced for *Impα5*, then differentiated with T3 for 24–72 h. In **(E)**, data for the myelin marker Cnp are from 72 h; other data are from 24 h. Note that differentiation markers are strongly reduced in *Impα5-*silenced cultures at both the RNA **(E)** and protein levels **(F,G)**. See also S6F Fig. **(H)** OLP cultures from *Klf6*^*fl/fl*^*Rosa26*^*fl/fl*^ mice were exposed to *Ad5CMVCre* or *Ad5CMV* control, then nucleofected with either an Impα5 expression construct or empty vector. Cultures were treated with 40 ng/ml T3 and harvested at 72 h. Notably, recombinant Impα5 significantly increased the proportion of cells expressing the differentiation marker O4. Recombinant Impα5 also partially rescued differentiation in Klf6-deficient cultures. Numbers in panels **(G,H)** refer to proportion of cells positive for the maturation marker per field at 20x magnification, for at least four fields per condition. Data are mean ± SEM. Statistics, **(B,E,G)** Student’s *t* test, **(H)** ANOVA plus Bonferroni post-test, *p < 0.05, ** *p* <0.01, *** *p* <0.001. Scalebars, (**C,G,H)** 20 μm. Data are representative of at least three independent studies. ChIP-seq data are available on the GEO website (http://www.ncbi.nlm.nih.gov/geo/) (Accession number GSE79245) and in S4 Table. Individual values for all other quantifications are in S1 Data.

We used this cohort as a starting point for analyses to identify the mechanism of action of Klf6 in the oligodendrocyte lineage. Notably, this work analyzed Klf6 interactions with upstream gp130-Stat3 signaling (Fig 5), and its interactions with potential downstream importin effectors (Fig 6). Collectively, this work identified a gp130-Klf-importin axis that regulates oligodendrocyte viability and differentiation.

### Klf6 Exerts Positive Feedback upon Upstream gp130-Stat3 Signaling

Multiple pieces of evidence supported interactions of Klf6 and upstream gp130-Stat3 signaling. In addition to data showing gp130-mediated Klf6 induction, ChIP-seq identified Klf6 binding to the gp130 promoter (Fig 5C), and RNA-seq revealed Klf6 regulation of multiple gp130 signaling components (Fig 5D, and see S1–S3 Tables). Together, these results suggested that pro-myelinating gp130-Stat3 effects are mediated in part via Klf6, and that Klf6 also exerts feedback upon this pathway (Fig 5E). Our studies further suggested the link between gp130-Stat3 and Klf6 was specific and not open to flexibility. In *Stat3-*silenced OLP cultures, Klf6 was Cntf-insensitive, and no compensatory sensitivity to T3 was detected (S5E Fig).

Analysis of Klf6 regulation of gp130-Stat3 signaling in vivo supported this hypothesis. Previous studies show that Stat3 expression is dependent upon and can be used as a marker of gp130 activity [46]. We therefore analyzed whether *Klf6* inactivation impacted Stat3 expression in *Olig1Cre*:*Klf6*^*fl/fl*^ mice. In control spinal cords, expression was strongest in Mnr^+^ motor neurons (S6A Fig), and lower-level activity was detectable more widely, including in Olig2^+^ cell nuclei (Fig 5F). Importantly, Stat3 was reduced in Olig2^+^ cells in *Olig1Cre*:*Klf6*^*fl/fl*^ samples, suggesting Klf6 regulates gp130 pathway activity in oligodendrocytes in vivo (Fig 5F).

### Conditional *Stat3* Inactivation In Vivo Produces Failure of Myelination and Defects in Klf6 Expression

If gp130-Stat3 signaling driving Klf6 expression is important in white matter formation, conditional *Stat3* inactivation should disrupt this program. To test this hypothesis, we generated conditional *Stat3* knockout mice by crossing a floxed *Stat3* allele [47] with *Olig1Cre*. Data were also validated using *PdgfracreER*^*TM*^, which, like *NG2creER*^*TM*^, produces inducible inactivation in OLP [48]. In *Olig1Cre*:*Stat3*^*fl/fl*^ and *PdgfracreER*^*TM*^:*Stat3*^*fl/fl*^ mice, we correlated changes in Klf6 activity with defects in oligodendrocyte development. We then compared the results of these analyses with data from mice with selective *Klf6* inactivation (Fig 5G–5M, compare with Figs 1 and 2).

Importantly, similar to conditional *Klf6* knockouts, mice with selective *Stat3* inactivation showed loss of maturing oligodendrocytes and myelination failure. At E12.5-E14.5, OLP numbers in *Olig1Cre*:*Stat3*^*fl/fl*^ spinal cords were similar to controls, and early differentiation events were normal (Fig 5G and 5H). However, differentiating cells were lost from E16.5 (Fig 5G–5I), and myelination was disrupted (Fig 5J and 5K). This loss occurred via apoptosis (S6B Fig). OLP numbers and proliferation were unaffected (Figs 5G and S6C). Notably, these effects were accompanied by a lineage-specific defect in expression of Klf6 or Klf6-expressing cells (Fig 5L).

Interestingly, *Olig1Cre*:*Stat3*^*fl/fl*^ embryos also displayed features not shared with *Klf6* mutants. We detected progressive loss of viability of ventral Mnr^+^ motor neurons, which have the same origin as ventral OLP [27], and this resulted in perinatal lethality (Figs 5K and S6D). These data suggested that Stat3 is required for both motor neuron and oligodendrocyte development, while Klf6 is only necessary for oligodendrocyte development. To selectively test the contribution to oligodendrocyte development, we examined the phenotype of *PdgfracreER*^*TM*^:*Stat3*^*fl/fl*^ mice, which undergo inducible recombination in OLP but not neurons [48]. *Cre* was induced at E12.5, and embryos were harvested at E18.5. *PdgfracreER*^*TM*^:*Stat3*^*fl/fl*^ embryos displayed no changes in Mnr^+^ neurons, but loss of differentiating oligodendrocytes and myelination deficits (Fig 5M).

Collectively, these data suggested that Klf6 and upstream gp130-Stat3 signaling are linked and play essential roles in CNS myelination. Inactivation of either in oligodendrocytes results in disruption of the other, associated with loss of differentiating oligodendrocytes and myelination failure.

### Klf6 Promotes Differentiation via Transactivation of the Importin Effector Impα5

Since our RNA-seq and ChIP-seq data identified direct Klf6 regulation of Impα5 (Fig 6A and S3 Table), the major importin in the adult CNS [41], we hypothesized that the gp130-Klf6 axis may use importins as important downstream effectors. Further analysis strongly suggested that Klf6 regulates oligodendrocyte development in part via transactivation of Impα5 (Figs 6 and S6).

We initially tested whether Klf6 regulation of Impα5 is selective, using qPCR in *Klf6-*silenced OLP, and iOL differentiated with T3 for 18 h (Fig 6B). The family of alpha importins function by binding transcription factors at nuclear localization sequences (NLS), and associating with importin-β subunits to translocate through the nuclear pore [41]. These studies confirmed that Impα5 was downregulated in both *Klf6*-silenced OLP and iOL cultures, and showed that other family members, including Impα1, Impα3, and Impβ1, were unaffected, thus demonstrating selectivity (Fig 6B). Examination of *Klf6*-silenced cultures exposed to pro-myelinating stimuli and treated with the caspase inhibitor Q-VD-OPh 2 μM confirmed that loss of Impα5 in these cultures was not due simply to apoptotic loss of Impα5-expressing cells (S6E Fig). Validating in vivo relevance, we detected reduced expression of Impα5 in the oligodendrocyte lineage in spinal cords of mice with conditional *Klf6* or *Stat3* inactivation (Fig 6C). Collectively, these findings were consistent with the hypothesis that the gp130-Klf6 axis uses selective control of Impα5 as a downstream effector mechanism to regulate oligodendrocyte development (Fig 6D).

### Impα5 Is Required for Oligodendrocyte Viability and Differentiation

To test whether disruption of normal importin expression limits oligodendrocyte differentiation and viability, we silenced *Impα5* in primary cultures, then differentiated with T3 for 24–72 h, and analyzed via confocal imaging, immunoblotting, and qPCR. Notably, silencing of *Impα5* produced a phenotype very similar to that seen following *Stat3* or *Klf6* inactivation, which was further compatible with known roles for importins in differentiation and viability [41,42]. *Impα5* silencing resulted in strong inhibition of differentiation, as demonstrated by reduced expression of myelin and maturation markers (Fig 6E–6G). Moreover, failure of differentiation was also associated with apoptosis of differentiating cells (S6F Fig).

If Klf6 regulation of Impα5 is functionally important in the context of early OLP differentiation, persistent expression of Impα5 might be expected to rescue, in part, the impact on differentiation of *Klf6* inactivation. To test this hypothesis, we used the viral vector *Ad5CMVCre* to inactivate *Klf6* in OLP cultures from *Klf6*^*fl/fl*^*Rosa26*^*fl/fl*^ mice. We then nucleofected the resulting Klf6-deficient cells and controls exposed to *Ad5CMV* control with either an Impα5 expression construct or empty vector. Cultures were then treated with 40 ng/ml T3, harvested at 72 h, immunostained for the differentiation marker O4, and subjected to confocal imaging. *Tomato* expression from the recombined *Rosa26*^*fl/fl*^ locus was used to confirm *Cre-*mediated recombination.

Notably, these experiments showed that expression of recombinant Impα5 in control OLP significantly increased the proportion of cells expressing differentiation markers. Moreover, and importantly, recombinant Impα5 partially rescued differentiation in Klf6-deficient cultures (Fig 6H). Thus, these studies confirmed a functional role for Impα5 in OLP differentiation, and suggested that it operates in the context of the gp130-Stat3-Klf6 axis.

Collectively, the findings of these experiments showed that importin activity is required for oligodendrocyte differentiation and that Impα5 overexpression potentiates differentiation. They further suggested that the gp130-Klf6 axis regulates oligodendrocyte development in part via Impα5-based control of nuclear trafficking.

## Discussion

This study identifies an essential role for the transcription factor Klf6 in CNS myelination and shows that it acts as a control point through which a pro-myelinating signal regulates oligodendrocyte development. Extrinsic cues are important regulators of CNS myelination [5], but the mechanisms by which they regulate viability, proliferation, and differentiation are incompletely characterized. Our findings place Klf6 within a novel axis driven by upstream gp130-Stat3 signaling, previously implicated as promoting survival and maturation in oligodendrocytes and other lineages [7,10,11,31,32]. Our data show that gp130/Stat3-driven Klf6 induction promotes differentiation, whereas mice with lineage-selective inactivation of *Klf6* or upstream Stat3 signaling show profound failure of CNS myelination, which results in tremor, ataxia, and death. Lineage specification and OLP proliferation and migration remain intact in these mice. The earliest events in differentiation occur normally, but subsequent steps in differentiation to the iOL stage are delayed, and terminal maturation fails and is associated with apoptosis. Compatible with the precedent set by gp130-Stat3 signaling, our studies further suggest that Klf6 plays distinct roles in both differentiation and viability. Pharmacologic inhibition of apoptosis in Klf6-deficient cultures does not restore differentiation. Interestingly, these findings contrast sharply with the lack of detectable developmental impact of inactivation of astrocytic *Klf6* in mice using an *mGfapCre* cassette, despite the fact that gp130 signaling is known to be important in astrocyte development and response to injury [49–51].

Members of the Klf/Sp transcription factor family fulfill diverse functions, in roles that are lineage- and time-dependent [13,52]. Our findings improve our understanding of their contributions to CNS development and molecular mechanisms of action. Klf6 has previously been studied as a tumor suppressor in prostate, ovary, and liver [18], and Klf4, Klf5, and Klf6 are also implicated as regulators of viability [13]. Our data now reveal that in the CNS, Klf6 regulates oligodendrocyte development and is driven by gp130-Stat3 signaling. This work further implicates the nuclear import protein Impα5 as an important, directly regulated Klf6 downstream effector. Interestingly, analysis of an external published RNA-seq database also reveals similar expression profiles for gp130, Stat3, Klf6, and Impα5, which in that dataset all progressively decrease during oligodendrocyte differentiation [29]. While our in vitro data suggest a critical requirement for gp130-Klf6-Impα5 signaling in early differentiation, the decreased activity of the axis in mature oligodendrocytes likely indicates a less critical role in maintenance of the mature, myelin-forming cell. It is possible that other members of both families may take over those roles following maturation.

While selective inactivation of importins is known to result in compensatory upregulation of other family members [42], our studies suggest that disruption of *Impα5* is sufficient to disrupt differentiation and viability. Furthermore, recombinant Impα5 expression significantly increases the number of differentiated cells in OLP cultures, and, importantly, recombinant Impα5 partially rescues differentiation in Klf6-deficient cultures. Cell-type-specific interactions between Klf family members and importins have only recently been identified [53]. Our data suggest that such an interaction, Klf6 transactivation of Impα5, may represent an important mechanism regulating oligodendrocyte survival and maturation, which is, moreover, sensitive to and feeds back upon an extrinsic gp130-mediated pro-myelinating signal. Importin canonical functions are to shuttle larger-sized cargo into the nucleus, with smaller factors entering passively through the nuclear pore complex [43]. Thus, we might expect various factors necessary for OLP differentiation to be size-selectively dependent on or independent of the Klf-importin axis. Accounting for the full range of importin regulation of transcription factor trafficking in oligodendrocytes will require co-immunoprecipitation and mass spectrometry analyses, with functional validation. While beyond the scope of the current report, such studies may produce a more complete understanding of the mechanisms regulating oligodendrocyte development.

Interestingly, while our data show that upstream gp130-Stat3 signaling drives Klf6 regulation of oligodendrocyte development, we also identify multiple components of the gp130 pathway as Klf6-regulated. Thus, our data suggest Klf6 participates in positive feedback regulation of a pro-myelinating pathway that drives its own expression. Furthermore, lineage-specific inactivation of *Stat3* produces a phenotype resembling conditional *Klf6* inactivation, including myelination failure, which is further associated with disruption of Klf6 and Impα5 expression, or loss of expressing cells within the oligodendrocyte lineage. Thus, our study validates Stat3 signaling as required for CNS myelination, and establishes Klf6 as a link between the upstream gp130-Stat3 pathway and a downstream importin effector. A secondary finding from our work also confirms the significance of Stat3 signaling in spinal cord motor neuron development, consistent with previous data [54]. This suggests a lineage-specific difference in neuronal versus oligodendrocyte development. We speculate that the role of Klf6 in oligodendrocytes may be filled in neurons by a different family member.

While Klf6 alone is not capable of initiating differentiation in our studies, its overexpression accelerates the rate of differentiation, as does Impα5 overexpression. Moreover, a basal level of each is required for differentiation, regardless of the triggering stimulus. Thus, these data suggest that the Klf6–Impα5 axis likely represents a component within the differentiation program, as opposed to an adaptor mechanism used by an extrinsic pro-myelinating signal. It is intriguing to speculate whether this mechanism may integrate more than one extrinsic input to the differentiation program, or whether it is specific for the gp130 pathway. Perhaps suggesting the latter, our data show that while Cntf readily activates the pathway, in contrast, T3 has no effect and, moreover, does not produce compensatory induction in the absence of a functional gp130-Stat3 pathway. Further studies are indicated to test whether Klf6 actions in oligodendrocyte development are under the control of just one pro-myelinating cue, or whether they may represent a point of convergence for additional extrinsic inputs.

In summary, our results connect an essential role for gp130/Stat3 signaling in CNS myelination, with importin-based control of nuclear trafficking as a downstream effector mechanism. Most notably, our findings implicate Klf6 as coordinating these events. Thus, our data reveal regulation of Klf6 as a mechanism via which an extrinsic signal promotes oligodendrocyte development and CNS myelination. Reconstructing early developmental processes responsible for the initiation and refinement of myelin formation may contribute to our understanding of the pathophysiology of neonatal, infantile, and pediatric leukoencephalopathies. Further studies will also determine whether reactivation of the gp130-Klf6 axis plays a significant role in promoting re-myelination in adults, in normal myelin turnover, or after injury, with the latter having implications for demyelinating disorders such as multiple sclerosis.

## Materials and Methods

### Mice

Work was approved by Institutional Animal Care and Use Committee at the Icahn School of Medicine at Mount Sinai under protocol numbers LA13-00029 and LA12-00235 and adhered to the American Veterinary Medical Association guidelines. Our institution has an Animal Welfare Assurance on file with the Office for Laboratory Animal Welfare. The Assurance number is A3111-01. *Olig1Cre* mice were generated by Q. Richard Lu, Charles Stiles (Harvard University, Massachusetts), and David Rowitch (University of California, San Francisco, California) [27]. *Klf6*^*fl*^ mice were generated and provided by Fréderic de Sauvage (Genentech, South San Francisco, California) and give a deletion/hypomorphic phenotype upon Cre recombination [26]. *Stat3*^*fl*^ mice were generated and provided by Shizuo Akira [47]. *NG2creER*^*TM*^ mice were generated by Akiko Nishiyama [30] and purchased from Jackson Laboratories (Bar Harbor, Maine). *PdgfracreER*^*TM*^ mice were generated and provided by Dwight Bergles [48]. *mGfapCre* mice were generated and provided by Michael V. Sofroniew (University of California Los Angeles) [28]. *Rosa26*^*CAG-tdTomato*^ mice were purchased from Jackson Laboratories (Bar Harbor, Maine). Genotyping was performed by PCR using primers in S6 Table. Conditional ablation of *Klf6* or *Stat3* in OLP in *NG2creER*^*TM*^ or *PdgfracreER*^*TM*^ embryos was induced by administration of 4-OH-tamoxifen (10 mg/mL) to pregnant mice at E12.5 and harvesting at E18.5 by euthanizing mothers with CO_2_ from compressed gas plus cervical dislocation, with the embryos removed by caesarean section and immediate decapitation prior to fixation. In the breeding programs for all mice, a strict set of humane endpoints was in place. Any mouse reaching any of these endpoints was immediately given an anesthetic overdose (barbiturate, 60 mg/kg ip), then received cardiac perfusion with either 4% paraformaldehyde or 4% glutaraldehyde for collection of tissue samples. Humane endpoints were: (1) weakness or paralysis such that the animal was unable to obtain food/water, (2) tremor severe enough to render the animal unable to move around the cage freely, (3) head tile or head turn associated with circling (animal unable to move freely), (4) depression/reduced level of activity to the point of failure to ingest food/water, (5) significant weight loss (>20% of body weight) associated with failure to eat/drink, (6) any other sign that the animal was unable to thrive in its environment. In addition, any animal showing any other complications (such as swelling, discharge, or pyrexia indicative of infection) was euthanized immediately using an anesthetic overdose. Mice were inspected at least once daily. In the initial breeding program for *Olig1Cre*:*Klf6*^*fl/fl*^ mice, we detected ataxia/tremor in the experimental genotype from 10 d of age. Additionally, one to two pups of the experimental genotype in the initial cohort died unexpectedly from a single grand mal seizure lasting 1–2 s. Once these phenotypes were detected, final breedings were subsequently planned such that mice were sacrificed prior to risk of suffering or mortality. In the breeding program for *Olig1Cre*:*Stat3*^*fl/fl*^ mice, in initial litters resulting from final matings, we detected pups of the experimental genotype dead immediately following birth. Thereafter, we used only developmental studies to characterize this line.

### OLP Cultures

Mouse OLP were isolated from P6-P7 C57BL6 mice and cultured as previously described [55]. Briefly, after removal of olfactory bulbs, brainstem, hippocampus, and meninges, cerebral cortices were isolated, dissociated, and resuspended in panning buffer. Single-cell suspensions were sequentially panned: twice on BSL1 plates for microglia depletion (15 min each), and then once on PDGFRa (CD140a, BD Bioscience) plates (45 min), and adherent cells were trypsinized and plated onto poly-D-lysine coated confocal dishes (Mat-Tek, Ashland, Massachusetts) in the proliferative factors PDGF-AA (10 ng/ml) and bFGF (20 ng/ml). After 24 h, cells were plated into differentiation medium containing 100 ng/ml Cntf or 40 ng/ml T3, or other factors or pharmacologic agents as specified in the text, and grown for up to 4 d. At plating, these cultures are >95% Olig2^+^Pdgfrα^+^Ng2^+^Cnp^-^Mbp^-^OLP. The immortalized cell line, Oli-neu [24], was grown in Sato medium + 1% horse serum (HS) and differentiated by withdrawal of HS for 24 h followed by the addition of T3 or Cntf at above concentrations.

### Growth Factors

PDGF-AA, bFGF, Lif, T3, and Cntf were purchased from Peprotech (Rocky Hill, New Jersey) and used at concentrations identified in the text.

### Inhibitors

The caspase inhibitor Q-VD-OPh and Jak inhibitor CAS457081-03-7 were obtained from EMD Bioscience and used at concentrations described in the text. For all experiments, growth factors were added on top of inhibitors, maintaining described concentrations.

### Transfection

For siRNA studies, 2.6 μg specific or nontargeting siRNA was nucleofected into OLP via electroporation using the Amaxa Basic Primary Neuron Nucleofector Kit (Lonza Cologne GmbH, Köln, Germany), protocol O-005, according to the manufacturer's instructions. In pilot studies for siKlf6, multiple siRNAs were used, corresponding to different regions of the target RNA, and specificity and efficiency were compared. The primary siRNA used to target Klf6 mRNA was J-043530-09 (GCAGGAAAGUUUACACGAA). Two additional siRNAs were used to assess specificity (J-043530-10: CUACAGAUUGUGCACGAAA; J-043530-12: GGACGAAUCCUGUAGGCUA). All other siRNA used in the text were ON-TARGETplus mouse siRNA-Smart Pool (GE Healthcare Dharmacon, Lafayette, Colorado) comprising four different siRNA targeting regions throughout specific genes of interest. Sham (transfection reagent alone) was used as an additional control. Extent and specificity of silencing were assessed by qPCR and immunoblotting. For expression constructs in OLP, cells were transfected using Amaxa Basic Primary Neuron Nucleofector Kit (Lonza Cologne GmbH, Köln, Germany), protocol O-005, with 1 μg expression vector pCMV6-AC-GFP containing GFP tagged Klf6, pCMV6-Entry containing Myc-DDK tagged Kpna1, or empty vector-GFP/DDK control (Origene, Rockville, Maryland). Expression was confirmed by immunostaining and confocal microscopy.

### Adenoviral Transfer In Vitro

OLP cultures from *Klf6*^*fl/fl*^*Rosa26*^*fl/fl*^ mice were performed as described above. Following expansion in proliferating media, OLPs were treated with either Ad5CMV-eGFP, for empty vector controls, or Ad5CMVCre-eGFP (Gene Vector Core, University of Iowa) to initiate Cre recombination (m.o.i = 22.5). After 4 h, cells were washed once in DMEM, nucleofected with Impα5 expression constructs or empty vector control as above, and incubated for 24 h in proliferating media prior to differentiation. Cells were counted the night prior to virus treatment to estimate m.o.i.

### Antibodies

Apc/CC-1 (mouse: Abcam, Cambridge, Massachusetts); β-actin (mouse: Santa Cruz Biotechnology, Santa Cruz, California); BrdU (chicken: Immunology Consultants, Newberg, Oregon; mouse: AbD Serotec, Raleigh, North Carolina); Cleaved caspase-3 (rabbit: Cell Signaling, Danvers, Massachusetts); Cnpase (mouse: Covance, Princeton, New Jersey); Gfap (rat: Life Technologies, Carlsbad, CA); GFP (chicken: Abcam, Cambridge, Massachusetts); Ki-67 (rabbit: Abcam, Cambridge, Massachusetts); Klf6 (mouse monocloncal, rabbit polyclonal: Santa Cruz Biotechnology, Santa Cruz, California; rabbit: LifeSpan Biosciences Inc, Seattle, Washington); Impα5 (rabbit: Proteintech, Chicago, Illinois), Mag (rabbit: Dr. Jim Salzer, New York University, New York); Mbp (mouse: Covance, Princeton, New Jersey; rabbit: Abcam, Cambridge, Massachusetts; rat: Millipore, Billerica, Massachusetts); Mnr2 (rabbit: Developmental Hybridoma Studies Bank, University of Iowa); NeuN (mouse: Millipore, Billerica, Massachusetts); Ng2/Cspg4 (rabbit: Developmental Hybridoma Studies Bank, University of Iowa); Nkx2.2 (mouse: Developmental Hybridoma Studies Bank, University of Iowa); O4 (mouse: Dr. Peter Davies, Albert Einstein College of Medicine, Bronx, New York); Olig2 (mouse, rabbit: Millipore, Billerica, Massachusetts); Pdgfrα (rabbit: Cell Signaling, Danvers, Massachusetts); Sox9 (rabbit: Millipore, Billerica, Massachusetts); Stat3 (rabbit, mouse: Cell Signaling, Danvers, Massachusetts); Stat3-p-Y705 (rabbit: Cell Signaling, Danvers, Massachusetts). Rabbit, mouse, goat IgG-HRP from Santa Cruz Biotechnologies, Santa Cruz, California. Secondary antibodies for immunofluorescence were conjugated to AlexaFluor 488, AlexaFluor568, or AlexaFluor 647 (Life Technologies, Carlsbad, California).

### Immunocytochemistry

Cultures were fixed with 4% paraformaldehyde for 10 min, rinsed three times with PBS, then incubated in blocking buffer (10% normal goat or donkey serum in PBS/Triton 0.3%) for 1 h at room temperature. Cells were incubated with primary antibodies for markers as specified in the text diluted in blocking buffer overnight at 4°C. For surface antigen staining, cells were blocked with 10% goat serum in PBS for 30 min and incubated with primary antibody. After rinsing three times with PBS/Triton 0.3%, samples were incubated with appropriate AlexaFluor conjugated secondary antibody, diluted 1:100 in blocking buffer for 1 h at room temperature. Following rinsing, cells were mounted using DAPI Fluoromount-G (SouthernBiotech, Birminhgam, Alabama) and photographed using a Leica Microsystems inverted confocal microscope (Buffalo Grove, Illinois) or Zeiss LSM 880 inverted confocal microscope (Jena, Germany). To assess proliferation, 10 μM BrdU (BD, San Diego, California) was added to cells 24 h prior to fixation. To assess apoptosis, immunohistochemistry using an antibody against cleaved caspase-3 was performed. To assess cell cycle exit, an index previously described [56] assessing the proportion of Ki67^-^BrdU^+^ cells to the total population of BrdU^+^ cells was used. Cells positive for all markers were quantified in *Z*-series stacks collected at x20 magnification with 1 μm on the Z-axis. Cells were counted in at least five stacks per condition per marker per time point in each experiment by a blinded observer using ImageJ software.

### Immunoblotting

Protein extracts were separated by sodium dodecyl sulfate-polacrylamide gel electrophoresis (SDS-PAGE) and transferred to onto PVDF membranes (Miliipore, Billerica, Massachusetts) using a buffer containing 25 mM Tris base, pH 8.3, 192 mM glycine, 20% (vol/vol) methanol for 1 h at 100 V at 4°C. Membranes were blocked for 1 h in 10% milk/0.1% Tween/TBS, then incubated overnight at 4°C with primary antibodies as described in the text. After incubation in primary antibody, membranes were rinsed with 0.1% Tween/TBS three times and subsequently incubated for 2 h at room temperature with secondary light-chain specific HRP-conjugated antibodies diluted 1:1000 in 10% milk/0.1% Tween/TBS. After rinsing, membranes were incubated with ECL (Thermo Scientific, Somerset, New Jersey) for 10 min and then revealed. For densitometry, unsaturated films were scanned using a Canon LiDE scanner (Lake Success, New York) and mean pixel density of each band measured using ImageJ software. Measurements were standardized to actin loading control, and fold change versus control was calculated.

### Immunohistochemistry

Frozen 25 μm sections of paraformaldehyde-perfused CNS were immunostained as described above and previously [57]. The following pre-treatments were used prior to blocking; for Mbp (SMI-99) or Stat3, sections were rinsed once in PBS, then incubated in 100% methanol at -20°C for 10 min and washed three time with PBS; for all other immunostainings, sections were soaked in 100°C citrate buffer pH 6 for 15 min, then let to cool in the buffer to room temperature and washed three time in PBS. Samples were then incubated in blocking buffer (10% normal goat or donkey serum in PBS/Triton 0.3%) for 1 h at room temperature. Cells were incubated with primary antibodies for markers as specified in the text and diluted in blocking buffer overnight at 4°C. After rinsing three times with PBS/Triton 0.3%, samples were incubated with appropriate AlexaFluor conjugated secondary antibody and diluted 1:100 in blocking buffer for 1 h at room temperature. Following rinsing, cells were mounted using DAPI Fluoromount-G (SouthernBiotech, Birminhgam, Alabama). Immunofluorescent stains for Klf6, Impα5, and Pdgfrα in embryonic and P1 tissue were accomplished via a signal amplification protocol (Perkin Elmer). Samples were examined using a Leica DMI or Zeiss LSM880 confocal microscope, and stacks were collected using 1–2 μm on the Z-axis and assembled into projections. For assessment of proliferation, mice received 10 mg/kg BrdU injection 1 h before fixation. To assess apoptosis, immunohistochemistry using an antibody against cleaved caspase-3 was performed. Cross-sectional Mbp^+^ or NeuN^+^ area and number of cells positive for lineage or stage-specific markers were measured in each stack using ImageJ software (NIH) by a blinded observer. For analyses of spinal cord, lumbar sections were analyzed and patterns confirmed in thoracic sections. Total Mbp^+^ or NeuN^+^ area per section was measured, or total number of positive cells per section counted, in the number of mice per genotype per time point stated in each figure legend. For other regions of the CNS (cerebellum, corpus callosum), positive area was measured (for Mbp and NeuN) or number of cells counted in at least 5 fields at 20x magnification per animal, in the number of mice per genotype per time point stated.

### Histopathology

Epoxy-embedded sections of glutaraldehyde-perfused corpus callosum, cerebellum, lumbar spinal cord, and optic nerve (1 μm) were stained with toluidine blue and photographed on a Zeiss Axioplan2 microscope. For electron microscopy, serial sections were placed on 200 mesh copper grids, contrasted with lead citrate and uranyl acetate, and scanned in a Hitachi HS600 microscope (Hitachi Inc., Pleasanton, California, at least five fields per animal, at least four mice per genotype per time point). For measurement of myelin thickness and G ratio, axon and fiber diameter were measured in at least ten random axons per field (at least five fields per condition) by a blinded observer using ImageJ. Myelin thickness was defined as (fiber diameter—axon diameter)/2, and G ratio as (axon diameter)/(fiber diameter).

### RNA Isolation and qPCR Analysis

RNA was isolated from cultures using the RNeasy Mini kit (QIAGEN) and quality was assessed using a Nanodrop spectrophotometer (Wilmington, Delaware). RNA was used in a 20 μl reverse transcription reaction, using SuperScript RT-PCR kit (Invitrogen). qPCR was performed using PerfeCTa SYBR Green FastMix (Quanta Biosciences) and an Applied Biosystem 7900HT Sequence Detection PCR System. The melting curve of each sample was measured to ensure product specificity. Data were normalized to internal controls Actin/Gapdh/Rps11 and analyzed using the Pfaffl ΔΔCt method. Primers for qPCR are presented in S6 Table.

### BeadArray

CNS (brain and spinal cord) of experimental *Olig1Cre*:*Klf6*^*fl/fl*^ mice and sex-matched *Klf6*^*fl/fl*^ littermate controls at postnatal day 1 (five per group per experiment) were dissected and snap frozen, total RNA was extracted using the RNeasy Mini kit (Qiagen), and quality was assessed by Nanodrop spectrophotometer. Total RNA was converted to cDNA and subsequently to biotin-labeled cRNA using a commercially available kit (Illumina, San Diego, California) according to the manufacturer’s instructions, and 1.5 μg of cRNA was hybridized to Illumina MouseWG-6 v1.1 expression BeadArray of 45,200 cDNA probes. Raw expression data were subjected to quantile normalization and low-intensity value filtering using a cutoff of 200 and then were log2 transformed (MatLab, MathWorks, Natick, Massachusetts). Differentially expressed genes were identified using unpaired two-tailed *t* test with False Discovery Rate (FDR) correction *p* < 0.05. Qualifying genes were subjected to hierarchical clustering using Cluster 3.0 software (Michael Eisen, UC Berkeley), and converted to a colorimetric format using Java Treeview (Alok Saldanha, Open Source Clustering Software). Data are available on the GEO website (http://www.ncbi.nlm.nih.gov/geo/) (Accession number GSE79245).

### ChIP and ChIP-seq Analysis

Chromatin was isolated from OLP and early iOL (4×10^6^ cells per sample). Cells were crosslinked in 1% formaldehyde for 7 min, and nuclear extraction and lysis were subsequently performed using sequential cell lysis (HEPES 5 mM pH8, KCl 85 mM, NP 40 0.5%, Protease Inhibitors [Sigma], PMSF 1 mM) and nuclear lysis buffer (50 mM Tris-HCl pH 8.1, 10 mM EDTA, 1% SDS, Protease Inhibitors [Sigma], PMSF 1 mM). Chromatin was sonicated using a Bioruptor (Diagenode) sonicator to produce chromatin with an average length of 200–400 base pairs. The size of DNA in the sheared chromatin fragments was tested by gel electrophoresis and Bioanalyzer to ensure proper fragment size. Immunoprecipitation was performed with 2 μg of anti-Klf6 antibody (polyclonal rabbit anti-Klf6, Santa Cruz, Santa Cruz, California) per unit OD_260_ readings. A total of four units of chromatin were used per condition. The DNA recovered from chromatin that was not immunoprecipitated was used as input. Chromatin that was immunoprecipitated with pre-blocked Staph A cells (Millipore, Billerica, Massachusetts) in the absence of primary antibody was used as a negative control. Following immunoprecipitation overnight, chromatin was incubated with Staph A cells for 14 min at room temperature, and samples were washed twice in Dialysis Buffer (2 mM EDTA, 50 mM Tris-HCl pH 8.0, 0.2% Sarkosyl) and four times in IP wash buffer (Tris-HCl 100 mM, pH 9.0, LiCl 500 mM, NP40 1%, Protease inhibitors, PMSF 1 mM) modified for polyclonal antibodies. Immunoprecipitated chromatin and input DNA were reverse crosslinked in elution buffer (50 mM NaHCO_3_, SDS 1%) with the addition of NaCl 0.2 M by heating at 67°C and shaking at 1300 rpm using a thermomixer (Eppendorf) overnight. DNA was then purified from the elution using phenol-chloroform-isoamylalcohol followed by an overnight ethanol precipitation at -20°C.

The input and ChIP samples were sequenced by Illumina HiSeq 2000. ChIP-seq was performed with two biological replicates per condition. Read sequences were generated by analyzing images and base calling using Illumina data analysis pipeline. After QC filtering by FASTX (http://hannonlab.cshl.edu/fastx_toolkit/), only reads with a quality score Q20 in at least 90% of bases were included for analysis. Reads from both input and ChIP samples were aligned to mouse reference genome (mm9) using Bowtie2 [58]. The peaks in the ChIP sample in reference to the input sample in OLP and iOLs were called from read alignments by MACS algorithm [59], then annotated with gene information, including distance to TSS and locations in gene function elements (intron, exon, UTR, upstream or downstream) from genome mapping information of RefSeq transcripts. Peaks within an interval of −15 kb to +5 kb to TSS of annotated genes were compared, and peaks with a maximum gap of 500 bp were considered overlapped. Alignment and coverage of ChIP-Seq data were visualized using Integrative Genomics Viewer (IGV) software [60], after input subtraction using methods described previously [61]. FDR-adjusted *p*-values of <0.05 were deemed significant. Data are available on the GEO website (http://www.ncbi.nlm.nih.gov/geo/) (Accession number GSE79245).

### RNA-seq Analysis

250ng of total RNA per sample were isolated from duplicate independent cultures of OLP, and early iOL after 18h of differentiation using the RNeasy Mini kit (QIAGEN). Libraries were constructed using the TruSeq RNA Sample Prep Kit (Illumina), and sequenced using an Illumina HiSeq 2000. Output files were subjected to quality control using FASTQC (http://www.bioinformatics.babraham.ac.uk/projects/fastqc/). All reported metrics including read quality score, adapter sequence contamination, and nucleotide frequency bias passed the suggested thresholds for all samples. Reads were analyzed using the Tuxedo suite [62], using the standard suggested pipeline with default parameters. Adapter-trimmed reads were mapped to the mouse genome (mm9) using Tophat. Transcript quantification was performed using CuffLinks [63], with expression normalized using CuffNorm and differential gene expression assessed with CuffDiff. FDR-adjusted *p*-values of <0.05 were deemed significant. RNA-seq data are available on the GEO website (http://www.ncbi.nlm.nih.gov/geo/) (Accession number GSE79245).

### Gene Ontology

Analysis to identify physiologic functions or diseases most relevant to sets of differentially expressed genes in RNA-seq and microarray studies was carried out using Ingenuity software (Ingenuity Systems, Redwood City, California), which uses Fisher’s exact test to calculate a *p*-value that the association between biological functions and a set of genes is due to chance alone.

### Statistics

For multiple comparisons, ANOVA plus Bonferroni post-test was used. Comparisons between two groups used Student's *t* test. In BeadArray studies, data were compared using unpaired two-tailed *t* test with False Discovery Rate correction. *p* < 0.05 was considered significant.

## Supporting Information

S1 ARRIVE Checklist ARRIVE guidelines checklist for reporting in vivo experiments. (PDF)

S1 Data Individual values used for quantitation in the text, figures, and supplementary materials. (XLSX)

S1 Fig Construction and technical validation of *Klf6* conditional knockout lines. Data presented here support findings in Fig 1. **(A)** QPCR analysis of Klf6 responses to the pro-myelinating factors Cntf (100 ng/ml), Lif (100 ng/ml), and T3 (40 ng/ml) in Oli-neu cells. Data extend results in Fig 1B. **(B)** To inactivate *Klf6* in oligodendrocyte lineage cells or astrocytes in vivo, mice with loxP sites flanking *Klf6* exons 2 and 3 (*Klf6*^*fl*^) were crossed with *Olig1Cre* or *mGfapCre* lines. The DNA constructs for each allele are illustrated in this panel. **(C)** Results of PCR genotyping of lines produced by crossing the alleles shown in panel **(B)**. In both lines, final matings generate the experimental and three control genotypes each at approximately 25% of total. **(D)** Confocal images of P14 spinal cords of *Olig1Cre*:*Klf6*^*fl/fl*^ and *mGfapCre*:*Klf6*^*fl/fl*^ mice and *Klf6*^*fl/fl*^ controls. Specificity and efficiency of inactivation in each line are confirmed by confocal imaging for lineage markers. The percentage of cells in each lineage that are Klf6^+^ is shown. In controls, Klf6 localizes to nuclei of oligodendrocytes and neurons and Gfap^+^ astrocytes (see Fig 1E). The upper four panels show that Klf6 and Olig2 overlap in oligodendrocyte lineage nuclei in *Klf6*^*fl/fl*^ controls, whereas Olig2^+^ cells in *Olig1Cre*:*Klf6*^*fl/fl*^ samples are Klf6 negative. Demonstrating specificity of inactivation, NeuN^+^ neurons (lower motor neurons illustrated) are Klf6 positive in both genotypes. The lower four panels demonstrate that conversely, in *mGfapCre*:*Klf6*^*fl/fl*^ samples, Klf6 is selectively lost from nuclei of Gfap^+^ astrocytes, but not from Olig2^+^ oligodendrocyte lineage cells. Data shown are representative of at least three independent studies in separate cultures **(A)**, or from at least three pups per genotype **(C,D)**. Data in panel **(D)** are presented as mean +/- SEM. Scalebars, **(D)** 5μm. Individual values are in S1 Data.
(TIF)

S2 Fig Myelination failure in *Olig1Cre*:*Klf6*^*fl/fl*^ spinal cord. Data shown complement results in Fig 1. **(A–D)** Confocal imaging analysis of lumbar spinal cords of P14 *Olig1Cre*:*Klf6*^*fl/fl*^ mice, *mGfapCre*:*Klf6*^*fl/fl*^ mice, and *Klf6*^*fl/fl*^ controls. Areas outlined in **(A)** are shown at higher magnification, inset. In contrast to controls and samples from mice with astrocyte-selective inactivation, myelin proteins are almost completely absent from spinal cords of *Olig1Cre*:*Klf6*^*fl/fl*^ mice **(A,B)**. Immunoreactivity for myelin proteins is normal in peripheral nerve roots (**A**, arrowed). No differences are seen in either genotype in expression patterns of the astrocyte marker Gfap or numbers of NeuN^+^ neurons **(C,D)**. **(E–H)** Light microscopy of resin-embedded P14 *Olig1Cre*:*Klf6*^*fl/fl*^ and *Klf6*^*fl/fl*^ spinal cord stained with toluidine blue. Areas 1–3 outlined in **(E)** are shown at increased magnification in **(F–G)**, and panel **(G)** is shown at high magnification in **(H)**. Myelin sheaths are almost completely absent from *Olig1Cre*:*Klf6*^*fl/fl*^ white matter tracts, and almost no small cells with round nuclei characteristic of oligodendrocytes are present. In **(F)**, compare unmyelinated *Olig1Cre*:*Klf6*^*fl/fl*^ CNS white matter (“c”) with normally myelinated peripheral nerve roots (“p”) in the same samples. **(I–K)** Electron micrographs of P14 **(I,J)** and P19 **(K)** *Olig1Cre*:*Klf6*^*fl/fl*^ and *Klf6*^*fl/fl*^ spinal cords, with higher magnification images inset. In contrast to compact myelin observed in controls (for example, **I**, left panel and inset), almost no myelin sheaths are present in *Olig1Cre*:*Klf6*^*fl/fl*^ CNS samples at P14 (**I**, right panel and inset, **J**) or closer to death at P19 **(K)**. No axonal pathology or degeneration is seen at either time point. Peripheral nerve roots of *Olig1Cre*:*Klf6*^*fl/fl*^ mice are normally myelinated **(J)**. Confocal imaging data shown are from lumbar sections of two to four mice per genotype. Thoracic sections showed the same findings. Electron microscopy data from P14 are representative of three mice per genotype, electron microscopy data from P19 are from two mice per genotype due to scarcity of samples and matched findings from P14 samples. Results are presented as mean ± SEM. Statistics, **(B,D)** ANOVA plus Bonferroni test, ***p < 0.001. Scalebars, **(A,C)** main panels 150 μm, **(A)** inset 15 μm. Magnifications, **(E)** x10, **(F,G)** x500, **(H)** x1000, **(I,K)** x5,000, inset x20,000, **(J)** x3,000. Individual values are in S1 Data.
(TIF)

S3 Fig Failure of myelination throughout the CNS in *Olig1Cre*:*Klf6*^*fl/fl*^ mice. Results are complementary to and extend data in Fig 1. **(A–F)** Analysis of P14 *Olig1Cre*:*Klf6*^*fl/fl*^, *mGfapCre*:*Klf6*^*fl/fl*^, and control *Klf6*^*fl/fl*^ cerebellum and corpus callosum. In contrast to results from the other two genotypes, myelin markers are almost completely absent from cerebellar white matter **(A,B)** and corpus callosum **(C,D)** in *Olig1Cre*:*Klf6*^*fl/fl*^ samples, and both tracts are thinner than normal (**A,C**, arrowheads). No changes are seen in neuronal numbers **(E,F)**. **(G–I)** Light and electron microscopy of P14 cerebellum **(G)** and optic nerves **(H,I)** from *Olig1Cre*:*Klf6*^*fl/fl*^ and *Klf6*^*fl/fl*^ control mice. In **(G)**, the area outlined in the upper panel is shown at higher magnification, below. Both tracts are unmyelinated in *Olig1Cre*:*Klf6*^*fl/fl*^ mice. In the lower panel, a rare myelinated fiber found in an *Olig1Cre*:*Klf6*^*fl/fl*^ sample is arrowed. Granule and Purkinje neurons in adjacent cerebellar grey matter are normal. Data shown are from two to four mice per genotype (for confocal imaging data) or three mice per genotype (for light/electron microscopy data). Quantitative data are presented as mean ± SEM. Statistics, **(B,D,E,F)** ANOVA plus Bonferroni test, **p < 0.01, *** *p* < 0.001. Scalebars, **(A,C)** 100 μm. Magnifications, **(G)** upper panel x500, lower x1,200, **(H)** x600, **(I)** x2,000. Individual values are in S1 Data.
(TIF)

S4 Fig *Klf6* inactivation in OLP using different *Cre* drivers produces convergent phenotypes. Results extend and complement findings shown in Figs 1, 2 and 3. **(A)** BeadArray transcriptional analysis of total CNS of P1 *Olig1Cre*:*Klf6*^*fl/fl*^ pups and sex-matched *Klf6*^*fl/fl*^ littermates, five per group. These data were used to generate the Ingenuity gene ontology analysis presented in Fig 1M. Data have been normalized and log_2_ transformed, and differentially expressed transcripts identified using unpaired two-tailed *t* test with False Discovery Rate correction. All transcripts with significant FDR-*corrected p*-values (Q values) <0.05 are shown. Results have been subjected to hierarchical clustering and are presented as numbered clusters in colorimetric format. There are significant differences in 60 probes corresponding to 51 annotated transcripts, all but one of which are reduced in *Olig1Cre*:*Klf6*^*fl/fl*^ CNS. Of these, 24 correspond to 18 genes previously reported as oligodendrocyte-expressed, shown in blue text. There are no changes in markers of neurons, astrocytes, or inflammatory cells. Note that transcriptional profiling of entire CNS data does not distinguish between changes in expression in living cells, versus loss of the cells that normally express the transcript. **(B)** Results of confocal imaging analysis comparing Olig2^+^ cell number in P14 spinal cords of *Olig1Cre*:*Klf6*^*fl/fl*^ conditional knockout mice, *Olig1Cre*:*Klf6*^*fl/+*^ heterozygotes and *Klf6*^*fl/fl*^ and *Klf6*^*fl/+*^ controls. Data complement Fig 2A–2D. While Olig2^+^ cell numbers are severely reduced in *Olig1Cre*:*Klf6*^*fl/fl*^ mice, numbers in heterozygotes are normal, matching those in controls. **(C,D)** Confocal and morphometric analysis of spinal cords of E18.5 *NG2creER*^*TM*^:*Klf6*^*fl/fl*^ embryos, in which *Klf6* inactivation has been inducibly targeted to OLP. See also Fig 2M. **(C)** Similar to results from *Olig1Cre*:*Klf6*^*fl/fl*^ mice, *NG2creER*^*TM*^:*Klf6*^*fl/fl*^ mice display selective loss of differentiating oligodendrocytes. In contrast, and again similar to *Olig1Cre*:*Klf6*^*fl/fl*^ mice, OLP numbers in *NG2creER*^*TM*^:*Klf6*^*fl/fl*^ embryos are identical to controls **(D)**. **(E)** qPCR in Oli-neu cells treated with 100 ng/ml Cntf or 40 ng/ml T3. Both produce comparable rates of differentiation as measured by expression of myelin proteins (Cnpase) and downregulation of OLP markers (Pdgfrα). In Cntf- but not T3-treated cultures, Klf6 upregulation precedes differentiation. **(F)** Densitometry measurements of immunoblotting data for Klf6, Cnpase, and Pdgfrα in primary mouse OLP differentiated with T3 and harvested at 24 h intervals. Actin was used as a loading control. Klf6 expression decreases during differentiation, preceding loss of the OLP marker Pdgfrα and upregulation of the myelin protein Cnpase. **(G)** Findings from primary OLP cultures in which *Klf6* has been silenced using siRNAs specific for different regions of its mRNA sequence (right panel). Sequence 1 corresponds to the siRNA used in the remainder of the manuscript. Cultures have been treated with Cntf 100 ng/ml for 2 h, then RNA harvested for qPCR. These findings complement Fig 3A–3H. Data are presented as mean ± SEM. Statistics, **(A)** unpaired two-tailed *t* test with False Discovery Rate correction, **(B)** one-way ANOVA plus Bonferroni test, **(C,D)** Student’s *t* test, **(E)** two-way ANOVA plus Bonferroni post-test, *p < 0.05, ** *p* < 0.01, *** *p* < 0.001. Results shown in **(B-D)** are from lumbar spinal cord sections. Data are representative of two to three independent studies in independent cultures, and two to four mice per genotype per time point. All data in **(A)** are available on the GEO website (http://www.ncbi.nlm.nih.gov/geo/) (Accession number GSE79245). Individual values for all other quantifications are in S1 Data.
(TIF)

S5 Fig Identification and validation of Klf6-dependent transcripts in oligodendrocyte lineage cells. Data complement findings in Fig 4 and S1–S3 Tables. **(A,B)** Silencing strategy for samples used in RNA-seq analysis. **(A)** Primary mouse OLP are isolated and expanded, then nucleofected with siRNA specific for *Klf6* or non-targeting (NT) control. *Klf6*-silenced OLP and controls are harvested following attachment, or after an additional 18 h exposure to T3 (40 ng/ml) or vehicle, prior to any changes in viability as assessed via caspase cleavage assay **(B)**. **(C)** Expanded dataset for qPCR validation of RNA-seq results from primary mouse OLP (red), iOL (blue), and shared genes (purple). See also Fig 4D. **(D)** Expanded dataset for sensitivity of validated differentially expressed transcripts to Cntf treatment (100 ng/ml) in Oli-neu cells. Colored areas indicate the time period before peak response of Klf6 to Cntf. Note also that some Klf6-dependent genes are Cntf-independent (Plxnb3 and Stmn3 illustrated). See also Fig 4E. **(E)** Results of qPCR analysis of primary mouse OLP subjected to *Stat3* silencing as described in Materials and Methods, followed by treatment with 40ng/ml T3 for up to 2 h. In *Stat3-*silenced OLP cultures, there is no compensatory sensitivity of Klf6 to T3 treatment. Results are presented as mean ± SEM. Statistics, Students *t* test **(B,C)**, two-way ANOVA plus Bonferroni test **(E)**, * *p* < 0.05, ** *p* < 0.01, *** *p* < 0.001. Data shown are representative of two to three independent studies in separate cultures. Individual values are in S1 Data.
(TIF)

S6 Fig Validation of the gp130-Klf-importin axis in oligodendrocyte lineage cells. Data complement results presented in Figs 5 and 6. **(A)** Confocal image of lumbar spinal cord of an E16.5 wild-type mouse embryo labeled for Stat3 and the marker of ventral horn motor neurons, Mnr. Immunoreactivity for Stat3 is ubiquitous, but is strongest within ventral Mnr^+^ motor neurons (arrowed). Stat3 also localizes less strongly to Olig2^+^ oligodendrocyte lineage cells (see Fig 5F). **(B–D)** Results of confocal imaging analysis of spinal cords from embryos with *Stat3* inactivation targeted to oligodendrocyte lineage cells (*Olig1Cre*:*Stat3*^*fl/fl*^), and *Stat3*^*fl/fl*^ littermate controls. As shown in Fig 5G–5K, these mice display loss of differentiating oligodendrocytes from E16.5 onwards. Panels presented here show that this loss occurs via apoptosis **(B)**. OLP proliferation is unaffected **(C)**. As illustrated in Fig 5K and unlike mice with *Klf6* inactivation driven by the same *Cre* cassette, *Olig1Cre*:*Stat3*^*fl/fl*^ embryos also display progressive loss of Mnr^+^ motor neurons **(D)**. **(E)** Confocal imaging analysis of *Klf6*-silenced and NT control mouse OLP pretreated with 2 μM caspase inhibitor Q-VD-OPh or vehicle 2 h, then exposed to T3 for 18 h. *Klf6*-silenced cultures treated with Q-VD-OPh 2 μM display loss of Impα5 expression, suggesting that loss of Impα5 in these cultures is not due simply to apoptotic loss of Impα5-expressing cells. **(F)** Results of confocal analysis of primary mouse OLP cultures silenced for *Impα5* and non-targeting control, differentiated via T3 for 72 h, then immunolabeled for cleaved caspase-3. Data complement findings in Fig 6E–6G, which show that induction of differentiation and myelin markers is strongly reduced in cultures with a defective Klf-importin axis. Results presented here confirm that failure of differentiation in these cultures is associated with increased apoptosis. Data are representative of at least three independent experiments in separate cultures, or at least three mice per genotype. Data shown in **(B–D)** are from lumbar sections. Scalebar, **(A)** 25 μm. Statistics, **(B–D)** two-way ANOVA plus Bonferroni test, **(F)** Student’s *t* test, **p < 0.01, ***p < 0.001. Individual values are in S1 Data.
(TIF)

S1 Table RNA-seq profiling of Klf6*-*regulated transcripts in OLP. Results shown here complement data in Figs 4 and S5 and in S2 and S3 Tables. Results of RNA-seq profiling of primary mouse OLP silenced for *Klf6* versus non-targeting control to identify Klf6-regulated transcripts, as described in the text and illustrated in S5 Fig. Data from two separate cultures have been filtered for statistically significant differential expression with FDR correction *p* < 0.05 (Q value, shown). Results from iOL are in S2 Table. Transcripts shared by both OLP and iOL are also shown in S3 Table. This analysis identifies 172 unique Klf6-regulated transcripts in OLP. Regulated transcripts are presented together with FPKM values, fold change, Q value, Unigene ID identifiers and PMID numbers for relevant references, and associated functions (summarized in Fig 4B). Results are color-coded using the scheme illustrated in Figs 4–6 for primary OLP (red) and shared transcripts (purple). Data are available on the GEO website (http://www.ncbi.nlm.nih.gov/geo/) (Accession number GSE79245).
(XLSX)

S2 Table RNA-seq identification of Klf6*-*regulated genes in iOL. Data here accompany findings in Figs 4 and S5 and in S1 and S3 Tables. RNA-seq profiling results from primary mouse iOL silenced for *Klf6* versus non-targeting control. Data from two separate cultures have been filtered for statistically significant differential expression with FDR correction *p* < 0.05 (Q value, shown). Results from OLP are in S1 Table. Transcripts shared by both OLP and iOL are also in S3 Table. This analysis identifies 51 unique Klf6-regulated transcripts in iOL. Regulated transcripts are presented together with FPKM values for each condition, fold change, Q value, Unigene ID identifiers and PMID numbers for relevant references, and associated functions (summarized in Fig 4B). Results are color-coded using the scheme illustrated in Figs 4–6, for primary iOL (blue) and shared transcripts (purple). Data are available on the GEO website (http://www.ncbi.nlm.nih.gov/geo/) (Accession number GSE79245).
(XLSX)

S3 Table RNA-seq profiling of Klf6*-*regulated transcripts shared by OLP and iOL. Findings here extend and complement results shown in Figs 4 and S5 and in S1 and S2 Tables. RNA-seq profiling data from primary mouse OLP and iOL silenced for *Klf6* versus non-targeting control to identify Klf6-regulated transcripts, as described in the text and illustrated in S5 Fig. Results from two separate cultures per condition have been filtered for statistically significant differential expression with FDR correction *p* < 0.05 (Q value). Full datasets from OLP and iOL are in S1 and S2 Tables, respectively. Transcripts shared by both OLP and iOL are shown here. This analysis identifies 40 Klf6-regulated transcripts shared by both OLP and iOL. Regulated transcripts are presented together with Unigene ID identifiers and PMID numbers for relevant references, and associated functions (summarized in Fig 4B). Results are color-coded using the scheme illustrated in Figs 4–6, in which shared transcripts are colored purple. Note that since FPKM values for each condition, fold change, and Q value are shown for all regulated transcripts in OLP and iOL in S1 and S2 Tables, they are not shown again here. Data are available on the GEO website (http://www.ncbi.nlm.nih.gov/geo/) (Accession number GSE79245).
(XLSX)

S4 Table Results of ChIP-seq screening for Klf6 binding to promoters of prospective effectors. Data shown here complement Fig 5 and illustrate results of ChIP-seq profiling of primary mouse OLP and iOL subjected to chromatin immunoprecipitation for Klf6 versus input as described in the text. Data from one of two representative studies are presented for both OLP and iOL, filtered for statistical significance with FDR correction *p* < 0.05 (Q value, shown), and for peak amplitude. Results presented include location and summit peak of chromatin binding, fold enrichment over input, distance from nearest transcription start site, and Q value. Data are also available on the GEO website (http://www.ncbi.nlm.nih.gov/geo/) (Accession number GSE79245). Results are color-coded by distance from known transcriptional start sites of annotated genes shown, from +5 kb to -15 kb. Genes also identified in RNA-seq experiments are shown in bold (see S1–S3 Tables). Integration of data from the two ChIP-seq studies filtered as described identifies 577 peaks of Klf6 chromatin binding called uniquely in OLP, 416 in iOL, and 337 peaks called in both populations.
(XLSX)

S5 Table Identification of Klf6-dependent genes that are directly Klf6*-*regulated. This table complements Figs 4 and 5 and S1–S4 Tables. It shows results of superimposition of RNA-seq profiling of OLP and iOL silenced for *Klf6* versus non-targeting control (from S1–S3 Tables and Fig 4), and Klf6 ChIP-seq profiling of the same cell types (from S4 Table and Fig 5). Overlay of the RNA-seq and ChIP-seq datasets identifies 20 genes as prospective directly-regulated Klf6-dependent effectors. Data are presented as gene names and associated functions, accompanied by Unigene ID identifiers and PMID numbers for relevant references. Results are color-coded using the scheme illustrated in Figs 4–6 and S1–S3 Tables.
(XLSX)

S6 Table Primer sequences for genotyping and qPCR. Additional details for the Materials and Methods section. This table shows primer sequences for genotyping of mouse lines and for qPCR for validation of RNA-seq data and maturation data presented in the manuscript.
(XLSX)

S1 Video Related to Fig 1. Tremor and ataxia in *Olig1Cre*:*Klf6*^*fl/fl*^ mice. Video sequence showing two representative P11 *Olig1Cre*:*Klf6*^*fl/fl*^ mutants (center and right), and a sex-matched *Klf6*^*fl/fl*^ littermate control (left). The mutants are smaller than the control and display tremor and ataxia. All *Olig1Cre*:*Klf6*^*fl/fl*^ pups display similar signs, which are progressive, and none survive beyond 21 days of age. Data are typical of results from each genotype (at least 50 pups per genotype). A still image from this sequence is illustrated in Fig 1G.
(MPG)

## Abbreviations

Apc: adenomatous polyposis coli
CNS: central nervous system
Impα5: importin-α5
iOL: immature oligodendrocyte
Klf6: Krüppel-like factor-6
mOL: mature oligodendrocyte
MS: multiple sclerosis
NLS: nuclear localization sequences
NT: non-targeting
OL: oligodendrocyte
OLP: oligodendrocyte progenitor
PNS: peripheral nervous system
qPCR: quantitative PCR
RNA-seq: RNA sequencing
Sox9: SRY-box transcription factor 9

## References

[1] McDonald WI, Sears TA. Effect of demyelination on conduction in the central nervous system. Nature 1969; 221: 182–183. 5782713 10.1038/221182a0

[2] Berger J, Moser HW, Forss-Petter S. Leukodystrophies: recent developments in genetics, molecular biology, pathogenesis and treatment. Curr Opin Neurol 2001; 14: 305–312. 11371752 10.1097/00019052-200106000-00007

[3] Frohman EM, Racke MK, Raine CS. Multiple sclerosis—the plaque and its pathogenesis. N Engl J Med 2006; 354: 942–955. 16510748 10.1056/NEJMra052130

[4] Chang A, Tourtellotte WW, Rudick R, Trapp BD. Premyelinating oligodendrocytes in chronic lesions of multiple sclerosis. N Engl J Med 2002; 346: 165–173. 11796850 10.1056/NEJMoa010994

[5] Fancy SP, Chan JR, Baranzini SE, Franklin RJ, Rowitch DH. Myelin regeneration: a recapitulation of development? Annu Rev Neurosci 2011; 34: 21–43. 10.1146/annurev-neuro-061010-113629 21692657

[6] Barres BA, Lazar MA, Raff MC A novel role for thyroid hormone, glucocorticoids and retinoic acid in timing oligodendrocyte development. Development 1994;120: 1097–1108. 8026323 10.1242/dev.120.5.1097

[7] Butzkueven H, Zhang JG, Soilu-Hanninen M, Hochrein H, Chionh F, et al LIF receptor signaling limits immune-mediated demyelination by enhancing oligodendrocyte survival. Nat Med 2002; 8: 613–619. 12042813 10.1038/nm0602-613

[8] Guardiola-Diaz HM, Ishii A, Bansal R. Erk1/2 MAPK and mTOR signaling sequentially regulates progression through distinct stages of oligodendrocyte differentiation. Glia 2012; 60: 476–486. 10.1002/glia.22281 22144101 PMC3265651

[9] Huang JK, Jarjour AA, Nait Oumesmar B, Kerninon C, Williams A, et al Retinoid X receptor gamma signaling accelerates CNS remyelination. Nat Neurosci 2011; 14: 45–53. 10.1038/nn.2702 21131950 PMC4013508

[10] Stankoff B, Aigrot MS, Noel F, Wattilliaux A, Zalc B, et al Ciliary neurotrophic factor (CNTF) enhances myelin formation: a novel role for CNTF and CNTF-related molecules. J Neurosci 2002; 22: 9221–9227. 12417647 10.1523/JNEUROSCI.22-21-09221.2002 PMC6758026

[11] Zhang J, Zhang Y, Dutta DJ, Argaw AT, Bonnamain V, et al Proapoptotic and antiapoptotic actions of Stat1 versus Stat3 underlie neuroprotective and immunoregulatory functions of IL-11. J Immunol 2011; 187: 1129–1141. 10.4049/jimmunol.1004066 21709156 PMC3164308

[12] Hall AK, Miller RH. Emerging roles for bone morphogenetic proteins in central nervous system glial biology. J Neurosci Res 2004; 76: 1–8. 15048925 10.1002/jnr.20019

[13] McConnell BB, Yang VW. Mammalian Kruppel-like factors in health and diseases. Physiol Rev. 2010; 90: 1337–1381. 10.1152/physrev.00058.2009 20959618 PMC2975554

[14] Nuez B, Michalovich D, Bygrave A, Ploemacher R, Grosveld F. Defective haematopoiesis in fetal liver resulting from inactivation of the EKLF gene. Nature 1995;375: 316–318. 7753194 10.1038/375316a0

[15] Segre JA, Bauer C, Fuchs E. Klf4 is a transcription factor required for establishing the barrier function of the skin. Nat Genet 1999;22: 356–360. 10431239 10.1038/11926

[16] Shindo T, Manabe I, Fukushima Y, Tobe K, Aizawa K, et al Kruppel-like zinc-finger transcription factor KLF5/BTEB2 is a target for angiotensin II signaling and an essential regulator of cardiovascular remodeling. Nat Med 2002;8: 856–863. 12101409 10.1038/nm738

[17] Takahashi K, Yamanaka S. Induction of pluripotent stem cells from mouse embryonic and adult fibroblast cultures by defined factors. Cell 2006;126: 663–676. 16904174 10.1016/j.cell.2006.07.024

[18] Bureau C, Hanoun N, Torrisani J, Vinel JP, Buscail L, et al Expression and Function of Kruppel Like-Factors (KLF) in Carcinogenesis. Curr Genomics 2009;10: 353–360. 10.2174/138920209788921010 20119532 PMC2729999

[19] Moore DL, Blackmore MG, Hu Y, Kaestner KH, Bixby JL, et al KLF family members regulate intrinsic axon regeneration ability. Science 2009;326: 298–301. 10.1126/science.1175737 19815778 PMC2882032

[20] Laub F, Lei L, Sumiyoshi H, Kajimura D, Dragomir C, et al Transcription factor KLF7 is important for neuronal morphogenesis in selected regions of the nervous system. Mol Cell Biol 2005;25: 5699–5711. 15964824 10.1128/MCB.25.13.5699-5711.2005 PMC1157008

[21] Fu H, Cai J, Clevers H, Fast E, Gray S, et al A genome-wide screen for spatially restricted expression patterns identifies transcription factors that regulate glial development. J Neurosci 2009;29: 11399–11408. 10.1523/JNEUROSCI.0160-09.2009 19741146 PMC2775518

[22] Dugas JC, Ibrahim A, Barres BA. The T3-induced gene KLF9 regulates oligodendrocyte differentiation and myelin regeneration. Mol Cell Neurosci 2012;50: 45–57. 10.1016/j.mcn.2012.03.007 22472204 PMC4441621

[23] Fu H, Qi Y, Tan M, Cai J, Takebayashi H, et al Dual origin of spinal oligodendrocyte progenitors and evidence for the cooperative role of Olig2 and Nkx2.2 in the control of oligodendrocyte differentiation. Development 2002;129: 681–693. 11830569 10.1242/dev.129.3.681

[24] Jung M, Kramer E, Grzenkowski M, Tang K, Blakemore W, et al Lines of murine oligodendroglial precursor cells immortalized by an activated neu tyrosine kinase show distinct degrees of interaction with axons in vitro and in vivo. Eur J Neurosci 1995; 7: 1245–1265. 7582098 10.1111/j.1460-9568.1995.tb01115.x

[25] Chen Y, Balasubramaniyan V, Peng J, Hurlock EC, Tallquist M, et al Isolation and culture of rat and mouse oligodendrocyte precursor cells. Nat Protoc 2007; 2: 1044–1051. 17546009 10.1038/nprot.2007.149

[26] Leow CC, Wang BE, Ross J, Chan SM, Zha J, et al Prostate-specific Klf6 inactivation impairs anterior prostate branching morphogenesis through increased activation of the Shh pathway. J Biol Chem 2009;284: 21057–21065. 10.1074/jbc.M109.001776 19494112 PMC2742870

[27] Lu QR, Sun T, Zhu Z, Ma N, Garcia M, et al Common developmental requirement for Olig function indicates a motor neuron/oligodendrocyte connection. Cell 2002; 109: 75–86. 11955448 10.1016/s0092-8674(02)00678-5

[28] Garcia AD, Doan NB, Imura T, Bush TG, Sofroniew MV. GFAP-expressing progenitors are the principal source of constitutive neurogenesis in adult mouse forebrain. Nat Neurosci 2004;7: 1233–1241. 15494728 10.1038/nn1340

[29] Zhang Y, Chen K, Sloan SA, Bennett ML, Scholze AR, et al An RNA-sequencing transcriptome and splicing database of glia, neurons, and vascular cells of the cerebral cortex. J Neurosci 2014; 34: 11929–11947. 10.1523/JNEUROSCI.1860-14.2014 25186741 PMC4152602

[30] Zhu X, Hill RA, Dietrich D, Komitova M, Suzuki R, et al Age-dependent fate and lineage restriction of single NG2 cells. Development 2011; 138: 745–753. 10.1242/dev.047951 21266410 PMC3026417

[31] Durant L, Watford WT, Ramos HL, Laurence A, Vahedi G, et al Diverse targets of the transcription factor STAT3 contribute to T cell pathogenicity and homeostasis. Immunity 2010;32: 605–615. 10.1016/j.immuni.2010.05.003 20493732 PMC3148263

[32] Hirano T, Ishihara K, Hibi M. Roles of STAT3 in mediating the cell growth, differentiation and survival signals relayed through the IL-6 family of cytokine receptors. Oncogene 2000;19: 2548–2556. 10851053 10.1038/sj.onc.1203551

[33] Stolt CC, Lommes P, Sock E, Chaboissier MC, Schedl A, et al The Sox9 transcription factor determines glial fate choice in the developing spinal cord. Genes Dev 2003;17: 1677–1689. 12842915 10.1101/gad.259003 PMC196138

[34] Whittington N, Cunningham D, Le TK, De Maria D, Silva EM. Sox21 regulates the progression of neuronal differentiation in a dose-dependent manner. Dev Biol 2015;397: 237–247. 10.1016/j.ydbio.2014.11.012 25448693 PMC4325979

[35] Stolt CC, Lommes P, Friedrich RP, Wegner M. Transcription factors Sox8 and Sox10 perform non-equivalent roles during oligodendrocyte development despite functional redundancy. Development 2004; 131: 2349–2358. 15102707 10.1242/dev.01114

[36] Stolt CC, Rehberg S, Ader M, Lommes P, Riethmacher D, et al Terminal differentiation of myelin-forming oligodendrocytes depends on the transcription factor Sox10. Genes Dev 2002;16: 165–170. 11799060 10.1101/gad.215802 PMC155320

[37] Fancy SP, Baranzini SE, Zhao C, Yuk DI, Irvine KA, et al Dysregulation of the Wnt pathway inhibits timely myelination and remyelination in the mammalian CNS. Genes Dev 2009; 23: 1571–1585. 10.1101/gad.1806309 19515974 PMC2704469

[38] Wang S, Sdrulla AD, diSibio G, Bush G, Nofziger D, et al Notch receptor activation inhibits oligodendrocyte differentiation. Neuron 1998;21: 63–75. 9697852 10.1016/s0896-6273(00)80515-2

[39] Kitamura K, Yanazawa M, Sugiyama N, Miura H, Iizuka-Kogo A, et al Mutation of ARX causes abnormal development of forebrain and testes in mice and X-linked lissencephaly with abnormal genitalia in humans. Nat Genet 2002;32: 359–369. 12379852 10.1038/ng1009

[40] Pierani A, Moran-Rivard L, Sunshine MJ, Littman DR, Goulding M, et al Control of interneuron fate in the developing spinal cord by the progenitor homeodomain protein Dbx1. Neuron 2001;29: 367–384. 11239429 10.1016/s0896-6273(01)00212-4

[41] Goldfarb DS, Corbett AH, Mason DA, Harreman MT, Adam SA. Importin alpha: a multipurpose nuclear-transport receptor. Trends Cell Biol 2004;14: 505–514. 15350979 10.1016/j.tcb.2004.07.016

[42] Yasuhara N, Shibazaki N, Tanaka S, Nagai M, Kamikawa Y, et al Triggering neural differentiation of ES cells by subtype switching of importin-alpha. Nat Cell Biol 2007;9: 72–79. 17159997 10.1038/ncb1521

[43] Stewart M. Molecular mechanism of the nuclear protein import cycle. Nat Rev Mol Cell Biol 2007;8: 195–208. 17287812 10.1038/nrm2114

[44] Finzsch M, Stolt CC, Lommes P, Wegner M. Sox9 and Sox10 influence survival and migration of oligodendrocyte precursors in the spinal cord by regulating PDGF receptor alpha expression. Development 2008;135: 637–646. 10.1242/dev.010454 18184726

[45] Zhang Y, Ni S, Huang B, Wang L, Zhang X, et al Overexpression of SCLIP promotes growth and motility in glioblastoma cells. Cancer Biol Ther 2015;16: 97–105. 10.4161/15384047.2014.987037 25511414 PMC4623355

[46] Ernst M, Jenkins BJ. Acquiring signalling specificity from the cytokine receptor gp130. Trends Genet 2004;20: 23–32. 14698616 10.1016/j.tig.2003.11.003

[47] Takeda K, Kaisho T, Yoshida N, Takeda J, Kishimoto T, et al Stat3 activation is responsible for IL-6-dependent T cell proliferation through preventing apoptosis: generation and characterization of T cell-specific Stat3-deficient mice. J Immunol 1998;161: 4652–4660. 9794394

[48] Kang SH, Fukaya M, Yang JK, Rothstein JD, Bergles DE. NG2+ CNS glial progenitors remain committed to the oligodendrocyte lineage in postnatal life and following neurodegeneration. Neuron 2010; 68: 668–681. 10.1016/j.neuron.2010.09.009 21092857 PMC2989827

[49] Nakashima K, Wiese S, Yanagisawa M, Arakawa H, Kimura N, et al Developmental requirement of gp130 signaling in neuronal survival and astrocyte differentiation. J Neurosci 1999;19: 5429–5434. 10377352 10.1523/JNEUROSCI.19-13-05429.1999 PMC6782325

[50] Haroon F, Drogemuller K, Handel U, Brunn A, Reinhold D, et al Gp130-dependent astrocytic survival is critical for the control of autoimmune central nervous system inflammation. J Immunol 2011;186: 6521–6531. 10.4049/jimmunol.1001135 21515788

[51] Okada S, Nakamura M, Katoh H, Miyao T, Shimazaki T, et al Conditional ablation of Stat3 or Socs3 discloses a dual role for reactive astrocytes after spinal cord injury. Nat Med 2006;12: 829–834. 16783372 10.1038/nm1425

[52] Moore DL, Apara A, Goldberg JL. Kruppel-like transcription factors in the nervous system: novel players in neurite outgrowth and axon regeneration. Mol Cell Neurosci 2011; 47: 233–243. 10.1016/j.mcn.2011.05.005 21635952 PMC3143062

[53] Kamikawa Y, Yasuhara N, Yoneda Y. Cell type-specific transcriptional regulation of the gene encoding importin-alpha1. Exp Cell Res 2011;317: 1970–1978. 10.1016/j.yexcr.2011.05.024 21664354

[54] Oppenheim RW, Prevette D, Yin QW, Collins F, MacDonald J. Control of embryonic motoneuron survival in vivo by ciliary neurotrophic factor. Science 1991; 251: 1616–1618. 2011743 10.1126/science.2011743

[55] Cahoy JD, Emery B, Kaushal A, Foo LC, Zamanian JL, et al A transcriptome database for astrocytes, neurons, and oligodendrocytes: a new resource for understanding brain development and function. J Neurosci 2008; 28: 264–278. 10.1523/JNEUROSCI.4178-07.2008 18171944 PMC6671143

[56] He Y, Dupree J, Wang J, Sandoval J, Li J, et al The transcription factor Yin Yang 1 is essential for oligodendrocyte progenitor differentiation. Neuron 2007; 55: 217–230. 17640524 10.1016/j.neuron.2007.06.029 PMC2034312

[57] Zhang Y, Argaw AT, Gurfein BT, Zameer A, Snyder BJ, et al Notch1 signaling plays a role in regulating precursor differentiation during CNS remyelination. Proc Natl Acad Sci U S A 2009;106: 19162–19167. 10.1073/pnas.0902834106 19855010 PMC2776461

[58] Langmead B, Salzberg SL. Fast gapped-read alignment with Bowtie 2. Nat Methods 2012;9: 357–359. 10.1038/nmeth.1923 22388286 PMC3322381

[59] Zhang Y, Liu T, Meyer CA, Eeckhoute J, Johnson DS, et al Model-based analysis of ChIP-Seq (MACS). Genome Biol 2008;9: R137 10.1186/gb-2008-9-9-r137 18798982 PMC2592715

[60] Thorvaldsdottir H, Robinson JT, Mesirov JP. Integrative Genomics Viewer (IGV): high-performance genomics data visualization and exploration. Brief Bioinform 2013;14: 178–192. 10.1093/bib/bbs017 22517427 PMC3603213

[61] Kharchenko PV, Tolstorukov MY, Park PJ. Design and analysis of ChIP-seq experiments for DNA-binding proteins. Nat Biotechnol 2008;26: 1351–1359. 10.1038/nbt.1508 19029915 PMC2597701

[62] Trapnell C, Roberts A, Goff L, Pertea G, Kim D, et al Differential gene and transcript expression analysis of RNA-seq experiments with TopHat and Cufflinks. Nat Protoc 2012;7: 562–578. 10.1038/nprot.2012.016 22383036 PMC3334321

[63] Roberts A, Pimentel H, Trapnell C, Pachter L. Identification of novel transcripts in annotated genomes using RNA-Seq. Bioinformatics 2011;27: 2325–2329. 10.1093/bioinformatics/btr355 21697122

