# Supplementary figures and images for: The Transcriptional Activator Krüppel-like Factor-6 Is Required for CNS Myelination
Source: PLoS Biol. 2016 May 23;14(5):e1002467. doi: 10.1371/journal.pbio.1002467 (PMC4877075; doi:10.1371/journal.pbio.1002467)

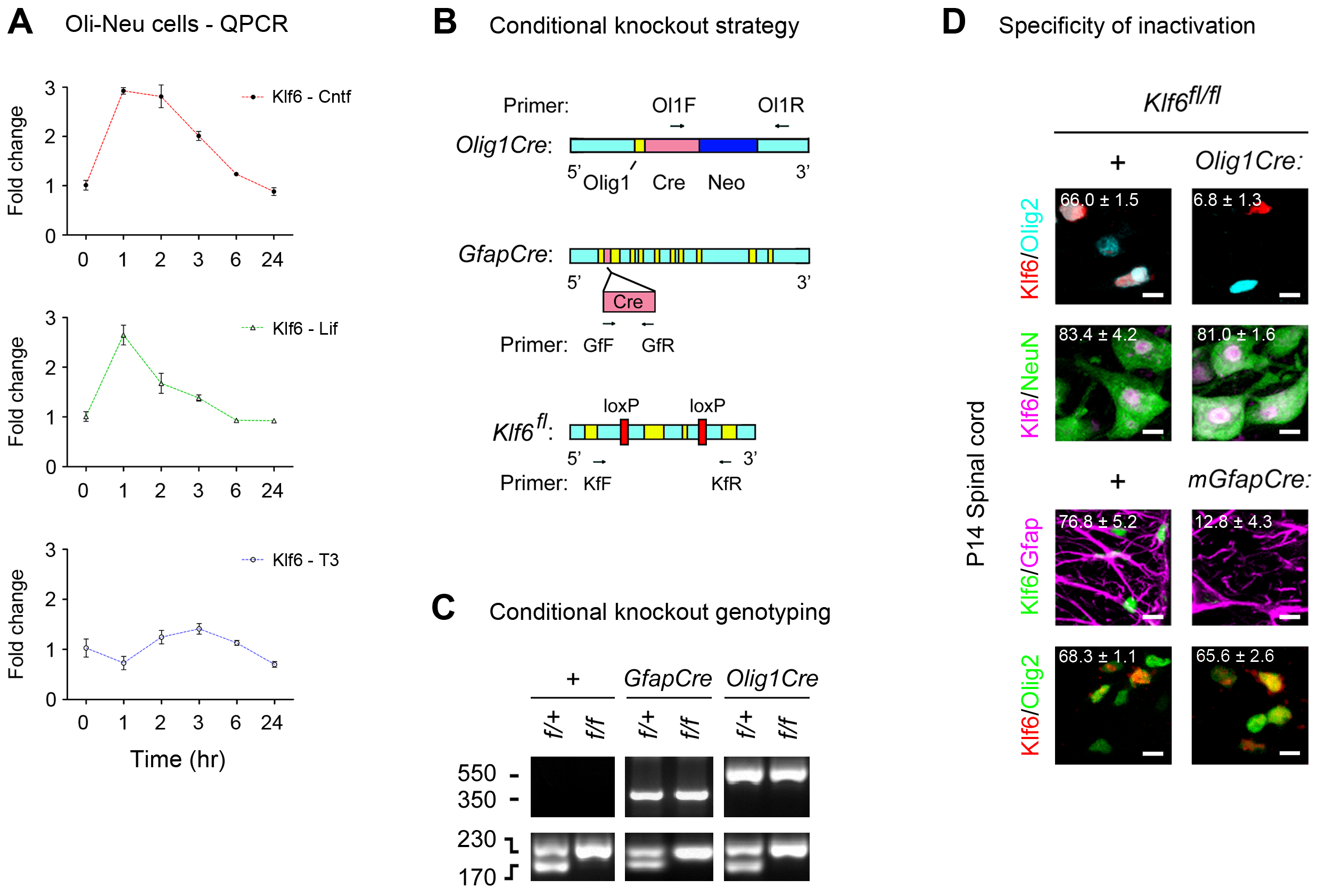

Supplement: S1 Fig — Data presented here support findings in Fig 1. (A) QPCR analysis of Klf6 responses to the pro-myelinating factors Cntf (100 ng/ml), Lif (100 ng/ml), and T3 (40 ng/ml) in Oli-neu cells. Data extend results in Fig 1B. (B) To inactivate Klf6 in oligodendrocyte lineage cells or astrocytes in vivo, mice with loxP sites flanking Klf6 exons 2 and 3 (Klf6fl) were crossed with Olig1Cre or mGfapCre lines. The DNA constructs for each allele are illustrated in this panel. (C) Results of PCR genotyping of lines produced by crossing the alleles shown in panel (B). In both lines, final matings generate the experimental and three control genotypes each at approximately 25% of total. (D) Confocal images of P14 spinal cords of Olig1Cre:Klf6fl/fl and mGfapCre:Klf6fl/fl mice and Klf6fl/fl controls. Specificity and efficiency of inactivation in each line are confirmed by confocal imaging for lineage markers. The percentage of cells in each lineage that are Klf6+ is shown. In controls, Klf6 localizes to nuclei of oligodendrocytes and neurons and Gfap+ astrocytes (see Fig 1E). The upper four panels show that Klf6 and Olig2 overlap in oligodendrocyte lineage nuclei in Klf6fl/fl controls, whereas Olig2+ cells in Olig1Cre:Klf6fl/fl samples are Klf6 negative. Demonstrating specificity of inactivation, NeuN+ neurons (lower motor neurons illustrated) are Klf6 positive in both genotypes. The lower four panels demonstrate that conversely, in mGfapCre:Klf6fl/fl samples, Klf6 is selectively lost from nuclei of Gfap+ astrocytes, but not from Olig2+ oligodendrocyte lineage cells. Data shown are representative of at least three independent studies in separate cultures (A), or from at least three pups per genotype (C,D). Data in panel (D) are presented as mean +/- SEM. Scalebars, (D) 5μm. Individual values are in S1 Data. (TIF) [file pbio.1002467.s003.tif]

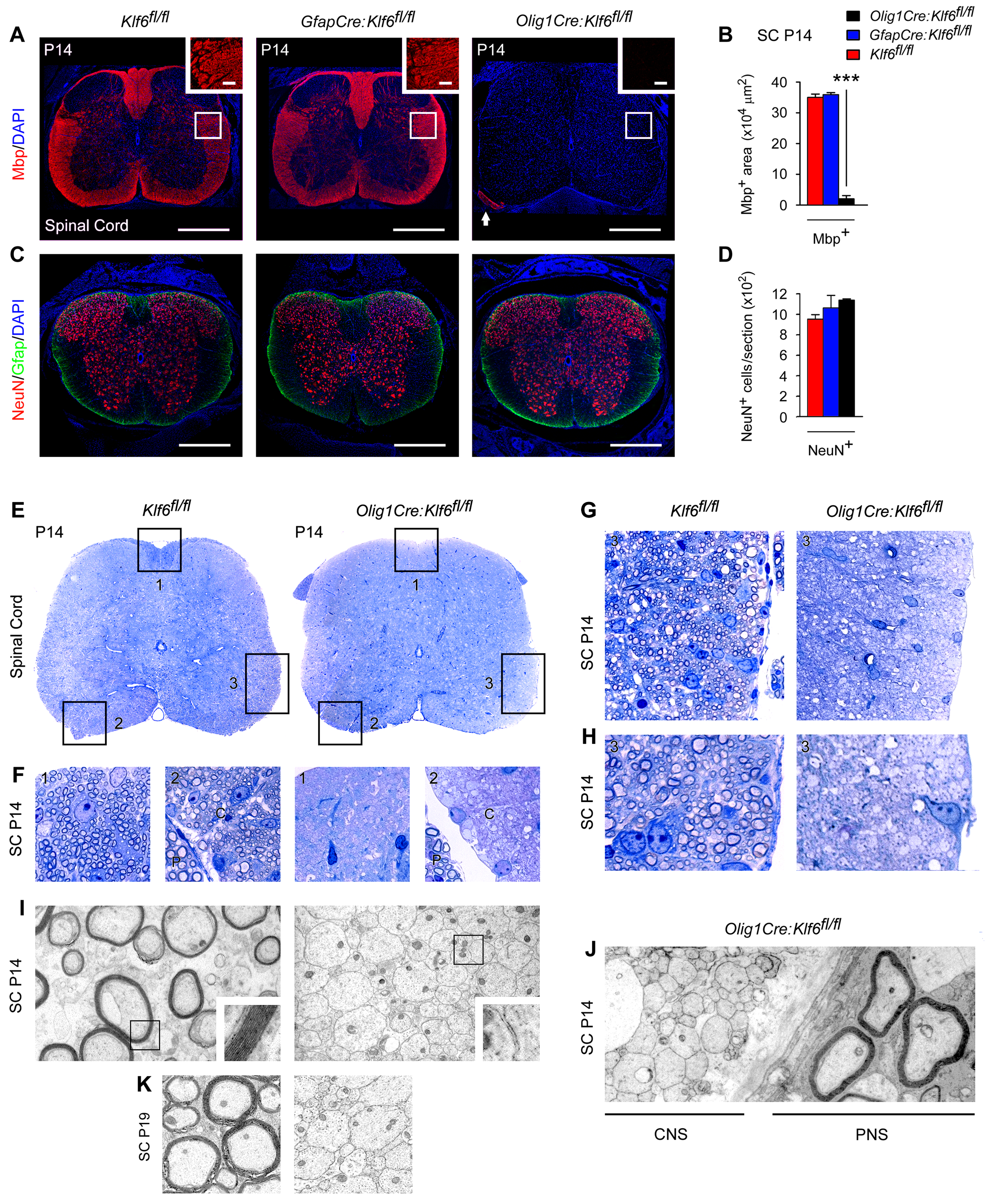

Supplement: S2 Fig — Data shown complement results in Fig 1. (A–D) Confocal imaging analysis of lumbar spinal cords of P14 Olig1Cre:Klf6fl/fl mice, mGfapCre:Klf6fl/fl mice, and Klf6fl/fl controls. Areas outlined in (A) are shown at higher magnification, inset. In contrast to controls and samples from mice with astrocyte-selective inactivation, myelin proteins are almost completely absent from spinal cords of Olig1Cre:Klf6fl/fl mice (A,B). Immunoreactivity for myelin proteins is normal in peripheral nerve roots (A, arrowed). No differences are seen in either genotype in expression patterns of the astrocyte marker Gfap or numbers of NeuN+ neurons (C,D). (E–H) Light microscopy of resin-embedded P14 Olig1Cre:Klf6fl/fl and Klf6fl/fl spinal cord stained with toluidine blue. Areas 1–3 outlined in (E) are shown at increased magnification in (F–G), and panel (G) is shown at high magnification in (H). Myelin sheaths are almost completely absent from Olig1Cre:Klf6fl/fl white matter tracts, and almost no small cells with round nuclei characteristic of oligodendrocytes are present. In (F), compare unmyelinated Olig1Cre:Klf6fl/fl CNS white matter (“c”) with normally myelinated peripheral nerve roots (“p”) in the same samples. (I–K) Electron micrographs of P14 (I,J) and P19 (K) Olig1Cre:Klf6fl/fl and Klf6fl/fl spinal cords, with higher magnification images inset. In contrast to compact myelin observed in controls (for example, I, left panel and inset), almost no myelin sheaths are present in Olig1Cre:Klf6fl/fl CNS samples at P14 (I, right panel and inset, J) or closer to death at P19 (K). No axonal pathology or degeneration is seen at either time point. Peripheral nerve roots of Olig1Cre:Klf6fl/fl mice are normally myelinated (J). Confocal imaging data shown are from lumbar sections of two to four mice per genotype. Thoracic sections showed the same findings. Electron microscopy data from P14 are representative of three mice per genotype, electron microscopy data from P19 are from two mice per genotyp [file pbio.1002467.s004.tif]

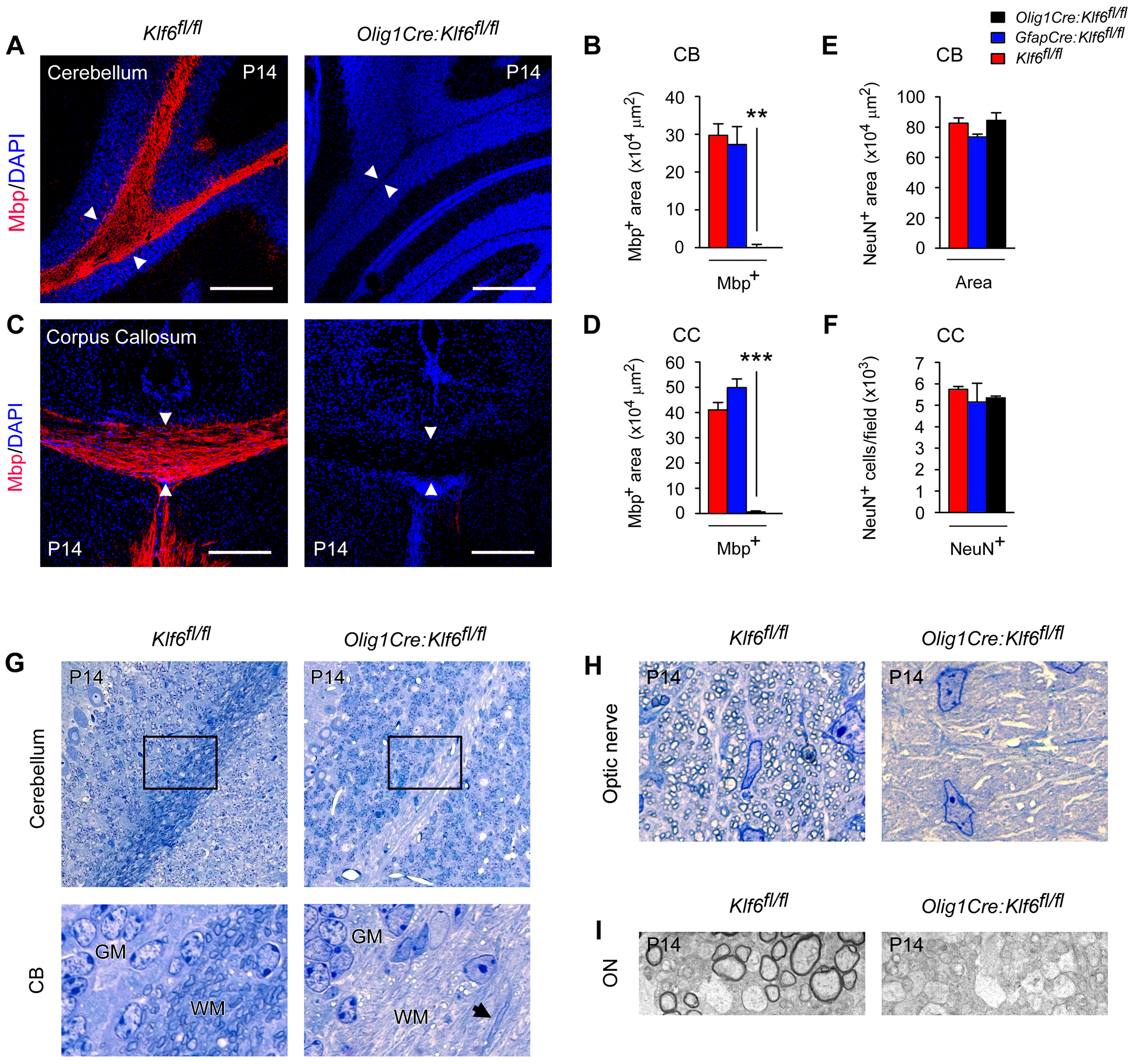

Supplement: S3 Fig — Results are complementary to and extend data in Fig 1. (A–F) Analysis of P14 Olig1Cre:Klf6fl/fl, mGfapCre:Klf6fl/fl, and control Klf6fl/fl cerebellum and corpus callosum. In contrast to results from the other two genotypes, myelin markers are almost completely absent from cerebellar white matter (A,B) and corpus callosum (C,D) in Olig1Cre:Klf6fl/fl samples, and both tracts are thinner than normal (A,C, arrowheads). No changes are seen in neuronal numbers (E,F). (G–I) Light and electron microscopy of P14 cerebellum (G) and optic nerves (H,I) from Olig1Cre:Klf6fl/fl and Klf6fl/fl control mice. In (G), the area outlined in the upper panel is shown at higher magnification, below. Both tracts are unmyelinated in Olig1Cre:Klf6fl/fl mice. In the lower panel, a rare myelinated fiber found in an Olig1Cre:Klf6fl/fl sample is arrowed. Granule and Purkinje neurons in adjacent cerebellar grey matter are normal. Data shown are from two to four mice per genotype (for confocal imaging data) or three mice per genotype (for light/electron microscopy data). Quantitative data are presented as mean ± SEM. Statistics, (B,D,E,F) ANOVA plus Bonferroni test, **p < 0.01, *** p < 0.001. Scalebars, (A,C) 100 μm. Magnifications, (G) upper panel x500, lower x1,200, (H) x600, (I) x2,000. Individual values are in S1 Data. (TIF) [file pbio.1002467.s005.tif]

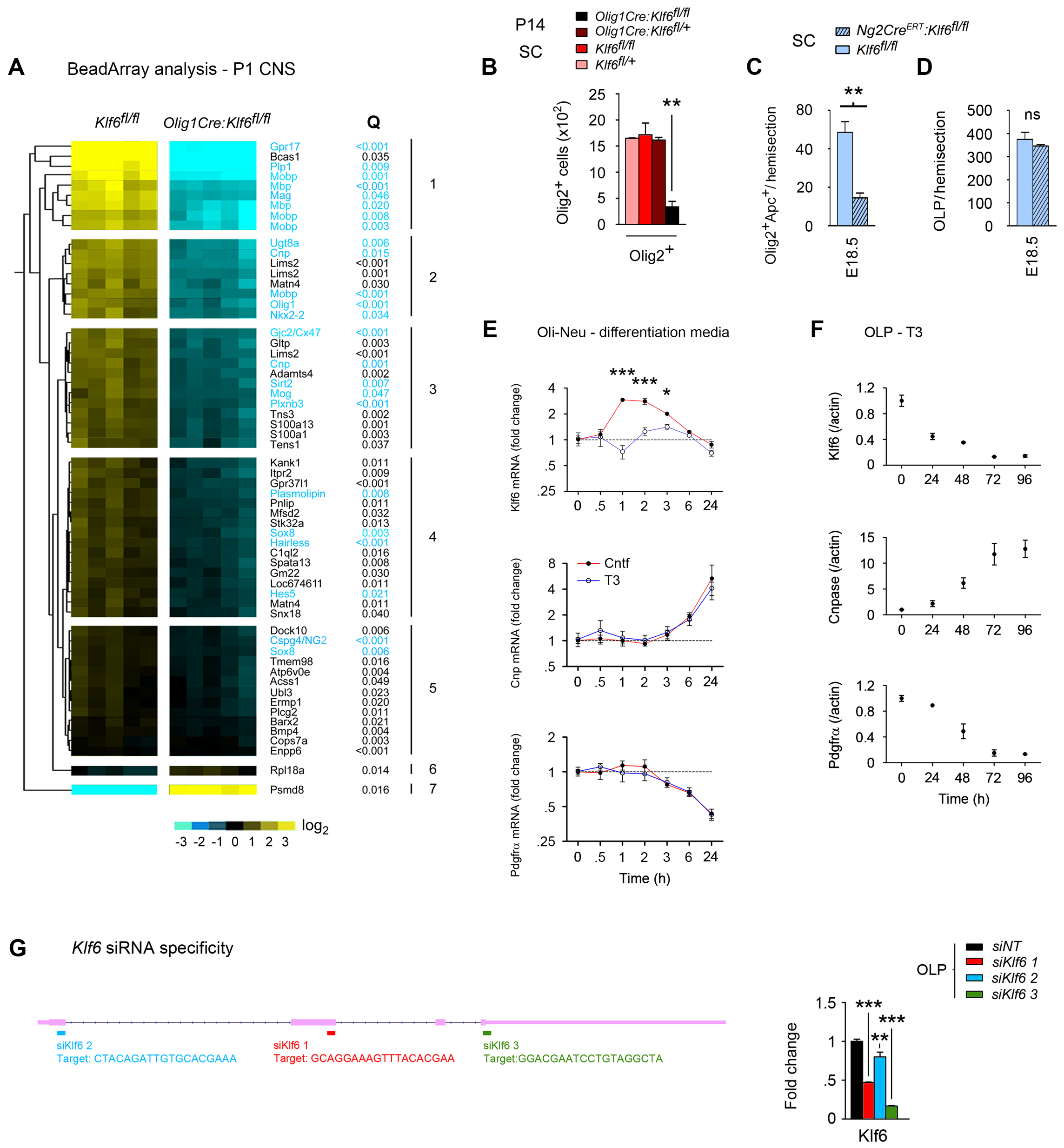

Supplement: S4 Fig — Results extend and complement findings shown in Figs 1, 2 and 3. (A) BeadArray transcriptional analysis of total CNS of P1 Olig1Cre:Klf6fl/fl pups and sex-matched Klf6fl/fl littermates, five per group. These data were used to generate the Ingenuity gene ontology analysis presented in Fig 1M. Data have been normalized and log2 transformed, and differentially expressed transcripts identified using unpaired two-tailed t test with False Discovery Rate correction. All transcripts with significant FDR-corrected p-values (Q values) <0.05 are shown. Results have been subjected to hierarchical clustering and are presented as numbered clusters in colorimetric format. There are significant differences in 60 probes corresponding to 51 annotated transcripts, all but one of which are reduced in Olig1Cre:Klf6fl/fl CNS. Of these, 24 correspond to 18 genes previously reported as oligodendrocyte-expressed, shown in blue text. There are no changes in markers of neurons, astrocytes, or inflammatory cells. Note that transcriptional profiling of entire CNS data does not distinguish between changes in expression in living cells, versus loss of the cells that normally express the transcript. (B) Results of confocal imaging analysis comparing Olig2+ cell number in P14 spinal cords of Olig1Cre:Klf6fl/fl conditional knockout mice, Olig1Cre:Klf6fl/+ heterozygotes and Klf6fl/fl and Klf6fl/+ controls. Data complement Fig 2A–2D. While Olig2+ cell numbers are severely reduced in Olig1Cre:Klf6fl/fl mice, numbers in heterozygotes are normal, matching those in controls. (C,D) Confocal and morphometric analysis of spinal cords of E18.5 NG2creERTM:Klf6fl/fl embryos, in which Klf6 inactivation has been inducibly targeted to OLP. See also Fig 2M. (C) Similar to results from Olig1Cre:Klf6fl/fl mice, NG2creERTM:Klf6fl/fl mice display selective loss of differentiating oligodendrocytes. In contrast, and again similar to Olig1Cre:Klf6fl/fl mice, OLP numbers in NG2creERTM:Klf6fl/fl embryos are identical to con [file pbio.1002467.s006.tif]

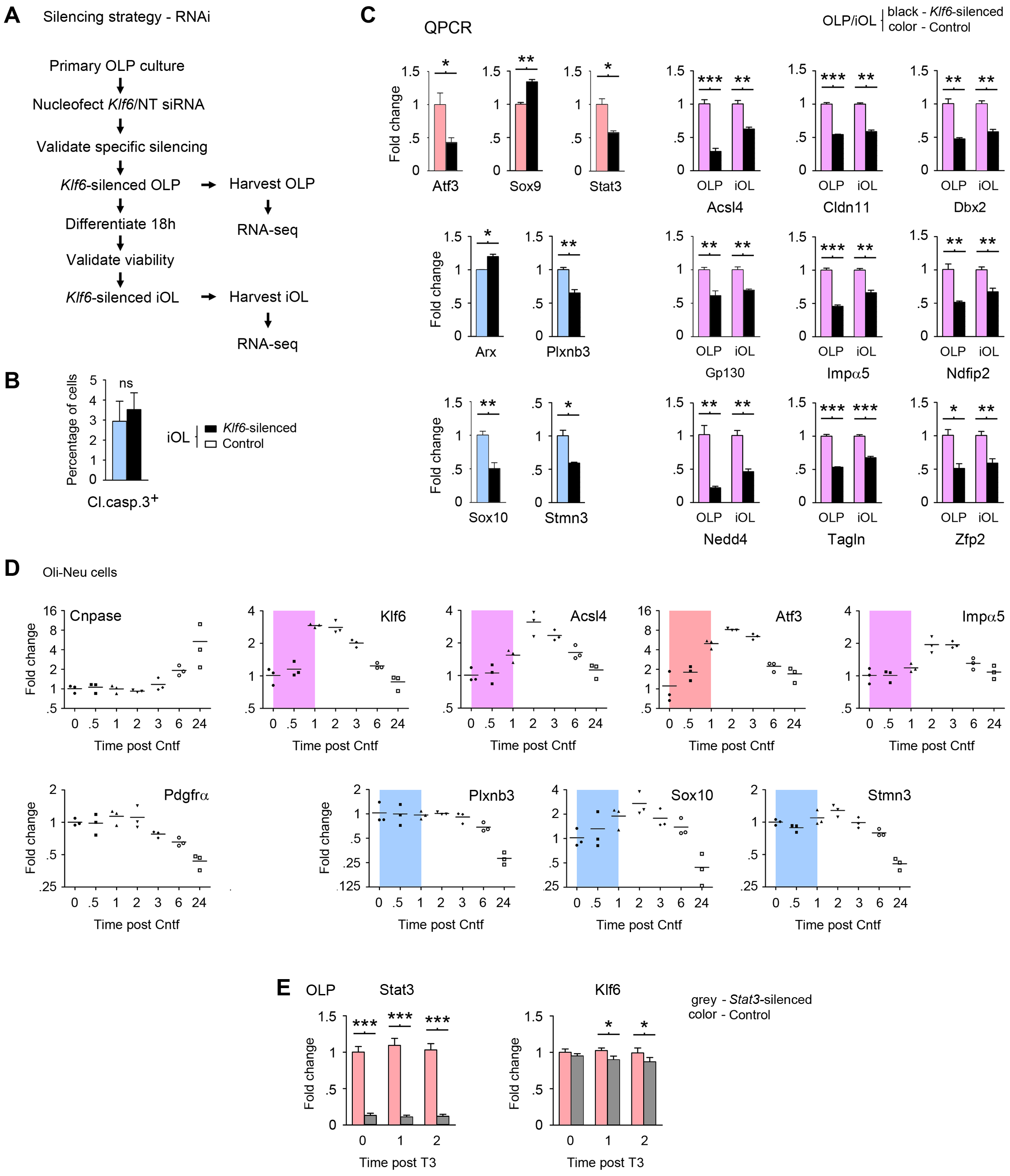

Supplement: S5 Fig — Data complement findings in Fig 4 and S1–S3 Tables. (A,B) Silencing strategy for samples used in RNA-seq analysis. (A) Primary mouse OLP are isolated and expanded, then nucleofected with siRNA specific for Klf6 or non-targeting (NT) control. Klf6-silenced OLP and controls are harvested following attachment, or after an additional 18 h exposure to T3 (40 ng/ml) or vehicle, prior to any changes in viability as assessed via caspase cleavage assay (B). (C) Expanded dataset for qPCR validation of RNA-seq results from primary mouse OLP (red), iOL (blue), and shared genes (purple). See also Fig 4D. (D) Expanded dataset for sensitivity of validated differentially expressed transcripts to Cntf treatment (100 ng/ml) in Oli-neu cells. Colored areas indicate the time period before peak response of Klf6 to Cntf. Note also that some Klf6-dependent genes are Cntf-independent (Plxnb3 and Stmn3 illustrated). See also Fig 4E. (E) Results of qPCR analysis of primary mouse OLP subjected to Stat3 silencing as described in Materials and Methods, followed by treatment with 40ng/ml T3 for up to 2 h. In Stat3-silenced OLP cultures, there is no compensatory sensitivity of Klf6 to T3 treatment. Results are presented as mean ± SEM. Statistics, Students t test (B,C), two-way ANOVA plus Bonferroni test (E), * p < 0.05, ** p < 0.01, *** p < 0.001. Data shown are representative of two to three independent studies in separate cultures. Individual values are in S1 Data. (TIF) [file pbio.1002467.s007.tif]

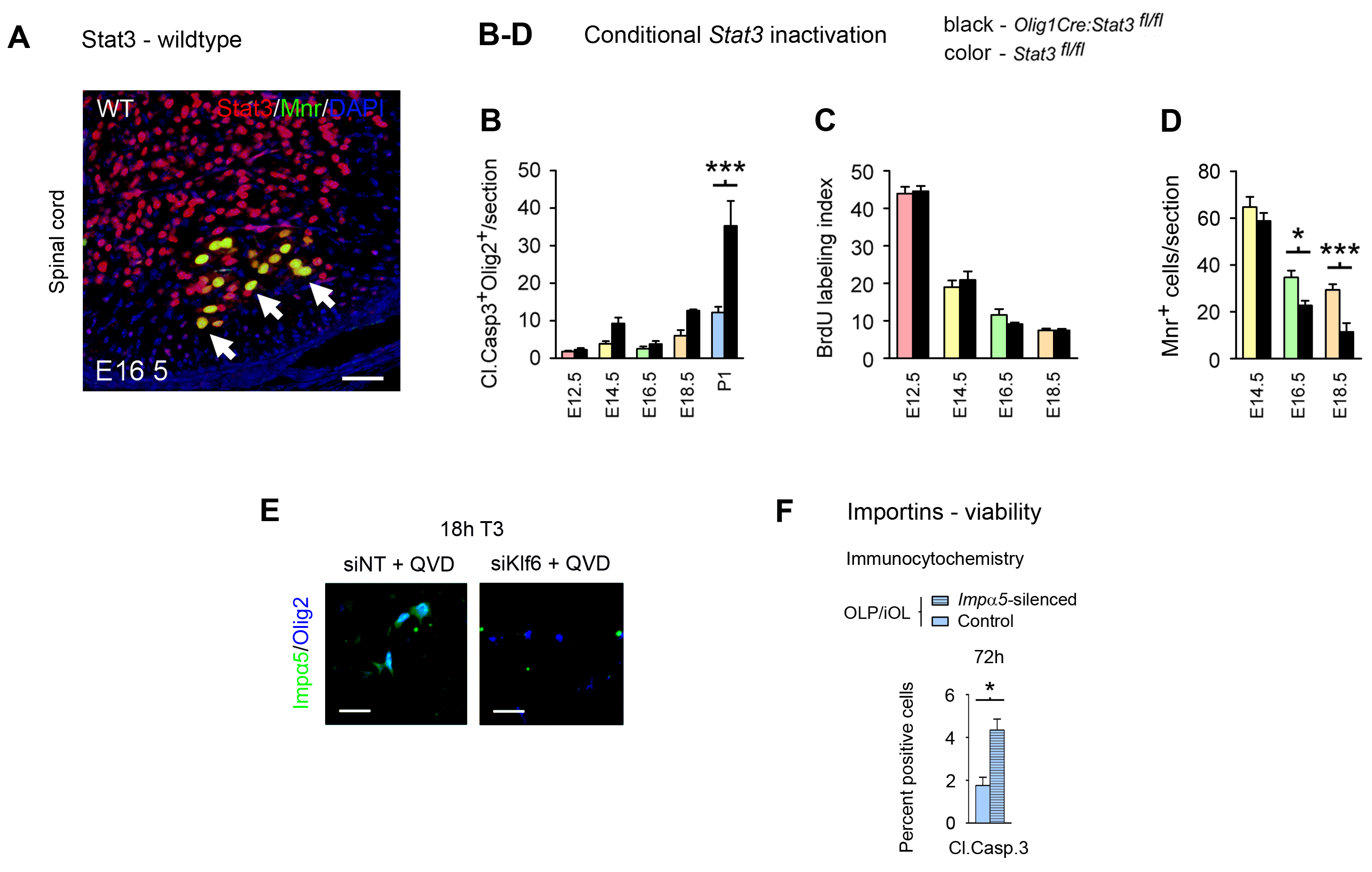

Supplement: S6 Fig — Data complement results presented in Figs 5 and 6. (A) Confocal image of lumbar spinal cord of an E16.5 wild-type mouse embryo labeled for Stat3 and the marker of ventral horn motor neurons, Mnr. Immunoreactivity for Stat3 is ubiquitous, but is strongest within ventral Mnr+ motor neurons (arrowed). Stat3 also localizes less strongly to Olig2+ oligodendrocyte lineage cells (see Fig 5F). (B–D) Results of confocal imaging analysis of spinal cords from embryos with Stat3 inactivation targeted to oligodendrocyte lineage cells (Olig1Cre:Stat3fl/fl), and Stat3fl/fl littermate controls. As shown in Fig 5G–5K, these mice display loss of differentiating oligodendrocytes from E16.5 onwards. Panels presented here show that this loss occurs via apoptosis (B). OLP proliferation is unaffected (C). As illustrated in Fig 5K and unlike mice with Klf6 inactivation driven by the same Cre cassette, Olig1Cre:Stat3fl/fl embryos also display progressive loss of Mnr+ motor neurons (D). (E) Confocal imaging analysis of Klf6-silenced and NT control mouse OLP pretreated with 2 μM caspase inhibitor Q-VD-OPh or vehicle 2 h, then exposed to T3 for 18 h. Klf6-silenced cultures treated with Q-VD-OPh 2 μM display loss of Impα5 expression, suggesting that loss of Impα5 in these cultures is not due simply to apoptotic loss of Impα5-expressing cells. (F) Results of confocal analysis of primary mouse OLP cultures silenced for Impα5 and non-targeting control, differentiated via T3 for 72 h, then immunolabeled for cleaved caspase-3. Data complement findings in Fig 6E–6G, which show that induction of differentiation and myelin markers is strongly reduced in cultures with a defective Klf-importin axis. Results presented here confirm that failure of differentiation in these cultures is associated with increased apoptosis. Data are representative of at least three independent experiments in separate cultures, or at least three mice per genotype. Data shown in (B–D) are from lumbar sections. Scalebar, (A) 25 μ [file pbio.1002467.s008.tif]
